# The Variant rs1784042 of the *SIDT2* Gene is Associated with Metabolic Syndrome through Low HDL-c Levels in a Mexican Population

**DOI:** 10.3390/genes11101192

**Published:** 2020-10-14

**Authors:** Guadalupe León-Reyes, Berenice Rivera-Paredez, Juan Carlos Fernandez López, Eric G. Ramírez-Salazar, Arnoldo Aquino-Gálvez, Katia Gallegos-Carrillo, Edgar Denova-Gutiérrez, Jorge Salmerón, Rafael Velázquez-Cruz

**Affiliations:** 1 Laboratorio de Genómica del Metabolismo Óseo, Instituto Nacional de Medicina Genómica (INMEGEN), Mexico City 14610, Mexico; greyes@inmegen.gob.mx; 2 Centro de Investigación en Políticas, Población y Salud de la Facultad de Medicina, Universidad Nacional Autónoma de Mexico (UNAM), Mexico City 04510, Mexico; bereriveraparedez7@gmail.com (B.R.-P.); jorge.salmec@gmail.com (J.S.); 3 Laboratorio de Genómica Computacional, Instituto Nacional de Medicina Genómica (INMEGEN), Mexico City 14610, Mexico; jfernandez@inmegen.gob.mx; 4 Consejo Nacional de Ciencia y Tecnología (CONACYT)-Laboratorio de Genómica del Metabolismo Óseo, Instituto Nacional de Medicina Genómica (INMEGEN), Mexico City 14610, Mexico; eramirez@inmegen.gob.mx; 5 Instituto Nacional de Enfermedades Respiratorias Ismael Cosío Villegas (INER), Mexico City 14080, Mexico; araquiga@yahoo.com.mx; 6 Unidad de Investigación Epidemiológica y en Servicios de Salud, Instituto Mexicano del Seguro Social (IMSS), Cuernavaca, Morelos 62000, Mexico; kgallegosc13@gmail.com; 7 Centro de Investigación en Nutrición y Salud, Instituto Nacional de Salud Pública (INSP), Cuernavaca, Morelos 62100, Mexico; edgar.denova@insp.mx

**Keywords:** *SIDT2* gene, HDL-c, metabolic syndrome, rs17120425, rs1784042, type 2 diabetes, Mexican population

## Abstract

The Mexican population has one of the highest prevalences of metabolic syndrome (MetS) worldwide. The aim of this study was to investigate the association of single-nucleotide polymorphisms (SNPs) with MetS and its components. First, we performed a pilot Genome-wide association study (GWAS) scan on a sub-sample derived from the Health Workers Cohort Study (HWCS) (*n* = 411). Based on GWAS results, we selected the rs1784042 and rs17120425 SNPs in the SIDT1 transmembrane family member 2 (*SIDT2*) gene for replication in the entire cohort (*n* = 1963), using predesigned TaqMan assays. We observed a prevalence of MetS in the HWCS of 52.6%. The minor allele frequency for the variant rs17120425 was 10% and 29% for the rs1784042. The SNP rs1784042 showed an overall association with MetS (OR = 0.82, *p* = 0.01) and with low levels of high-density lipoprotein (HDL-c) (odds ratio (OR) = 0.77, *p* = 0.001). The SNP rs17120425 had a significant association with type 2 diabetes (T2D) risk in the overall population (OR = 1.39, *p* = 0.033). Our results suggest an association of the rs1784042 and rs17120425 variants with MetS, through different mechanisms in the Mexican population. Further studies in larger samples and other populations are required to validate these findings and the relevance of these SNPs in MetS.

## 1. Introduction

Metabolic syndrome (MetS) is characterized by a set of metabolic factors that increase the risk of cardiovascular diseases (CVD), type 2 diabetes (T2D) and atherosclerosis [1,2]. According to the Adult Treatment Panel III (ATP III) criteria, in the Mexican population, the prevalence of MetS is 41.6%, one of the highest worldwide [3].

Genetic and environmental factors contribute to the pathophysiology of MetS [4,5]. Family and twin studies have provided the initial evidence for the heritability and co-occurrence of the metabolic traits. MetS heritability has been reported between 13% and 30% and for some individual metabolic components can be as high as 50% [4,5,6]. Several Genome-wide association studies (GWAS) have been performed to identify MetS-related single nucleotide polymorphisms (SNPs) considering independent components of MetS as quantitative traits [7,8,9]. In view of MetS as a binary phenotype, several GWAS have identified numerous loci influencing its presence. Most variants associated to MetS have been located within or near genes regulating lipid metabolism and seem to be relevant for the genetic background of MetS [10,11,12]. Specifically, genetic variants are of great interest when SNPs have different frequencies between populations, because it could lead to differences in gene expression [13]. To date, much of the genetic variation remains unexplained. Therefore, the search for genetic variants associated with development or exacerbation of this syndrome are of great interest.

GWAS in Asian populations [14,15] have shown that the SIDT1 transmembrane family member 2 (*SIDT2*) gene, encodes a SIDT2-lysosomal membrane protein and is associated with plasma triglyceride (TG) levels. Moreover, Moon et al. reported an association between *SIDT2* and MetS and its components, specifically with high density lipoprotein (HDL-c) and TG levels [16]. Recently, a meta-analysis conducted in a Korean population found that the SNP rs1784042 in *SIDT2* gene was associated with total cholesterol (TC) levels [17].

Recently, *SIDT2* has been related to lipid and glucose metabolism. Several investigations carried out in *Sidt2-/-* mice showed that the absence of this gene generates an increase in the TG and free fatty acids (FFA) levels in serum [18,19]. However, it could be involved in several functions, such as maintenance of the integrity of membrane and digestion and transport of lysosomal-products [20]. Furthermore, it may participate in the re-localization of cholesterol among organelles [21]. Its absence could cause an alteration of the autophagy-related lipid degradation pathway and an accumulation of autophagosomes which impair lipid metabolism [18]. In addition, the knock out mice model showed an impaired glucose tolerance due insulin secretion dysfunction, suggesting that *SIDT2* may be also involved in glucose metabolism [22,23].

Mexico is a middle-income country and Mexicans represent a unique population with a distinct genetic background, diet and sedentary lifestyle compared to other populations [24]. Moreover, Mexicans are more prone to suffer hypertriglyceridemia, glucose intolerance and obesity compared to Caucasians [25,26]. Many of such metabolic deregulations are extremely severe and have an earlier age of onset in Mexicans than in Caucasians [27,28]. Moreover, Mexicans have an augmented body fat mass, a greater intra-abdominal subcutaneous adipose tissue and greater accumulation of ectopic fat compared to Caucasians, resulting in an enhanced risk for MetS and CVD [3]. These reasons indicate that the Mexican population has a significant genetic predisposition to develop MetS.

Previously, we carried out a pilot GWAS in Mexican-Mestizo postmenopausal women (*n* = 411), which represent a subsample of the Health Workers Cohort Study (HWCS) over 61 years of age. In that study, we identified SNPs significantly associated with several metabolic traits [29,30]. The relative low frequency of some functional variants of *SIDT2* in several populations, provides a unique opportunity to assess association with clinical/metabolic traits, in admixed populations, such as the Mexican-Mestizo. The aim of this study was to investigate the association of the variants rs1784042 and rs17120425 of the *SIDT2* gene with MetS and its individual components in a cohort of Mexican Health Professionals. To the best of our knowledge, this study provides new and relevant information about the association between the rs1784042 and rs17120425 variants with MetS, through lowering HDL-c levels, in a Mexican population. With this information, we can contribute to the genetic knowledge involved in the MetS, which is a worldwide health problem.

## 2. Materials and Methods

### 2.1. Study Population

We performed a pilot GWAS and a candidate gene study in Mexican-Mestizo subjects from the HWCS. The details of the study design, methodology and participants’ baseline characteristics have been described previously [31].

For the current analysis, we included data from 2086 individuals who were invited to participate in the second measurement period of the HWCS (2010 and 2012) and from whom DNA and serum samples were available. We excluded individuals < 18 years (*n* = 85) and missing genotype data for both SNPs (*n* = 38). After these exclusions, a total of 1963 individuals (aged 18–92 years) were included in the analysis.

This research was performed in accordance with the Declaration of Helsinki. The study protocol and informed consent form were approved by the Research and Ethics Committee from the Instituto Mexicano del Seguro Social (IMSS, by its Spanish acronym) (No. 12CEI 09 006 14). All participants signed an informed consent form.

### 2.2. Demographic, Anthropometric and Clinical Measurements

Demographic data were obtained from a self-reported questionnaire [31]. The procedures for anthropometric and biochemical measurements were carried out as described previously [30,31]. MetS diagnosis was based on Adult Treatment Panel (ATP) III criteria according to American Heart Association/National Heart Lung and Blood Institute (referred as AHA/NHLBI) [3]. Postmenopausal women were defined as being 45 years of age or older and having experienced 12 months without a menstrual period. Family group was defined as two or more related individuals.

### 2.3. Sample Genotyping and Selection of SNPs for Validation

#### 2.3.1. Discovery Phase

In the first stage, a pilot GWAS scan was performed on 411 unrelated postmenopausal women, a sub-sample from the HWCS. DNA samples were genotyped using the Infinium HumanCytoSNP-12 DNA v2.1 chip, (Illumina Inc., San Diego, CA, USA), the inclusion thresholds executed: missing rate per person and SNPs were of 95%, we excluded markers that did not meet Hardy–Weinberg test at *p* value < 5 × 10^−6^ significance threshold, we removed SNPs with minor allele frequency (MAF) < 1%, we conducted an identity by descent (IBD) analysis to verify the absence of relatedness between individuals in this study. To determine the samples’ sex (female) we calculated X chromosome inbreeding (homozygosity) by the F estimate of PLINK software; we converted Linkage Pedigree format files to Variant Call Format (VCF) using PLINK v1.9. The markers imputation was performed with a Michigan Imputation Server (Minimac4 method) using a Haplotype Reference Consortium (HRC) panel (Version r1.1 2016), the phasing selection was Eagle v2.3 and Ad Mixed American (AMR) as the supported reference panel and finally we selected the quality Control and Imputation mode. A total of 7.2 million imputed SNPs from the HRC panel [32] in 396 women with an overall call rate of 99.68%, genotype imputation quality minimum of 0.4 and MAF > 1%, were used for further analysis. The pilot GWAS with TC, Low-Density Lipoprotein cholesterol (LDL-c), HDL-c, glucose and TG serum levels was carried out using the EPACS Genome Analysis tool as follows: we performed a quantitative linear Wald Test model using residual inverse normal transformation of age, body mass index (BMI) and two ancestry principal components (ancestry correction). The study design and methodology are described in detail elsewhere [29,30]. Briefly, the median age of the cohort was 61 years (55–68), median BMI was 27.5 (25.3–34.3) and 68.2% had MetS.

#### 2.3.2. Replication Phase

The present study is part of a large-scale genetic study to identify genetic variants affecting bone mineral density (BMD) and related quantitative metabolic traits in the Mexican population [29,30].

In the replication phase, derived from the results of the pilot GWAS, we selected two SNPs located in the *SIDT2* gene: the rs1784042, because its *p*-value in the discovery phase was *p* = 0.006 and it is located in a region previously associated with MetS and other metabolic traits. We also selected the missense variant p.*V636I* (rs17120425), based on: (1) proximity to the variant rs1784042 (~2473 bp), (2) no linkage disequilibrium (LD) with the variant rs1784042 (r^2^ = 0.10 and D’= 0.77) based on the Mexican Ancestry population from Los Angeles, California (MXL) and (3) differences in MAFs between populations (according to the 1000 genomes project, MAFs 0–6%). Both SNPs were genotyped using predesigned TaqMan assays (Applied Biosystems), in the entire sample, including the individuals from the first stage. The previous association of genes (*n* = 29) and their reported variants (*n* = 46) for MetS and related metabolic traits, were obtained from the National Human Genome Research Institute–European Bioinformatics Institute (NHGRI-EBI) GWAS Catalog [33] and a PubMed search.

### 2.4. Statistical Power of the Study

We calculated the power of the study using Quanto software (Department of Preventive Medicine, University of Southern California, Los Angeles, CA, USA) [34]. A log-additive model of inheritance for allele frequencies in the range from 0.06 to 0.10 and odds ratios (ORs) in the range from 0.80 to 1.40 for rs17120425 and the allele frequencies in the range from 0.28 to 0.30 and ORs in the range from 0.70 to 1.40 for rs1784042, derived from this study, the 1000 genomes project and the literature, were used. Prevalence of disease was taken in the range from 42% to 53% at a significance level of 0.05.

### 2.5. Conditional and Haplotype Association Analysis

To evaluate if the the two selected variants in the SIDT2 gene were independent, we performed a conditional analysis in the replication stage data employing a logistic regression model. Age, sex, family group and identified SNPs genotypes were included as covariables in the model using STATA software, version 14.0 (StataCorp LP, College Station, TX, USA). Haplotype-based association analysis of SIDT2 SNPs rs17120425 and rs1784042 was carried out using a logistic regression model adjusting for age, sex and family group in the replication phase data using Haploview [35] and STATA software, version 14.0 (StataCorp LP, College Station, TX, USA).

### 2.6. Statistical Analysis

The Hardy–Weinberg Equilibrium (HWE) was tested on each of the study groups using the chi-square test. The descriptive analysis of the demographic and clinical characteristics was stratified by genotype of both SNPs in each parameter. Furthermore, we estimated the prevalence of MetS and its components by age and sex. To determine the differences between groups, a chi square test was applied for the categorical variables and a Dunn’s test for the continuous variables (e.g., age, BMI). Genetic association analyses between rs17120425 and rs1784042 with the MetS-traits were carried out in the total population with adjustment for age, sex and family group. The association was modeled using multivariable linear and logistic regression. Additionally, the analysis was stratified by gender and menopausal status. Statistical analyses were performed using STATA software, version 14.0 (StataCorp LP, College Station, TX, USA). A *p* value < 0.025 (Bonferroni adjustment, α/n) was considered statistically significant.

## 3. Results

### 3.1. Demographic Data of the Study Population

This study included a total of 1963 participants from the HWCS; 70% were women and 30% were men. We observed that the men group had higher levels of overweight, waist circumference (WC), smoking, blood pressure (BP), fasting glucose, TC, TG and lower HDL-c levels than women (*p* < 0.05). The obesity, body fat proportion and LDL-c levels were higher in women (*p* < 0.05) (Table 1).

### 3.2. Prevalence of MetS and Its Components by Gender and Age Groups

**Figure 1:**
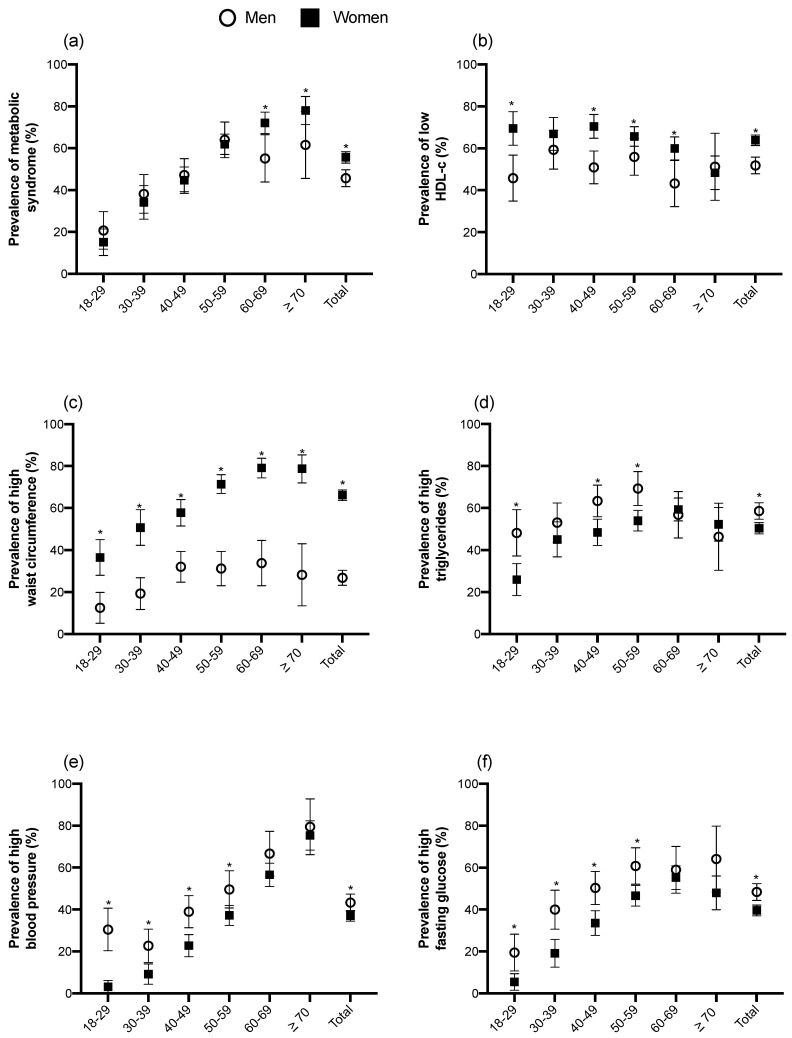
Prevalence of MetS and its components in different age groups stratified by gender. (**a**) Prevalence of MetS by The National Cholesterol Education Program (NCEP)—Adult Treatment Panel III (ATPIII); (**b**) prevalence of low (high-density lipoprotein HDL-c); (**c**) prevalence of high waist circumference; (**d**) prevalence of high triglycerides; (**e**) Prevalence of high blood pressure and (**f**) prevalence of high fasting glucose. (*) *p* value < 0.05. Lines represent the 95% confidence intervals.

The prevalence of MetS and its components were stratified by gender and age (Figure 1 and Supplementary Table S1). The overall prevalence of MetS in the HWCS was 52.6% (95%, Confidence Interval (CI) 50.4–54.8). Women had a higher prevalence of MetS than males (55.6 vs. 45.7%, *p* < 0.001; Figure 1a). Low HDL-c levels (Figure 1b) and high WC (Figure 1c) were more frequently observed in women than in men (63.9% vs. 51.8%, *p* = 0.0001 and 66.1% vs. 26.8%, *p* < 0.001, respectively) (Supplementary Table S1).

The overall prevalence of hypertriglyceridemia was of 53.1%, it was higher in men than women in all age groups (58.2% vs. 50.9%, *p* = 0.0028) (Figure 1d). Elevated BP prevalence was 38.9% overall; this was higher in men than women (43.3% vs. 37%, *p* = 0.010). Interestingly, in younger groups (≤30 years of age), men had 9.5 times higher prevalence of elevated BP than women within the same age range (30% vs. 3.17%, *p* < 0.001) (Figure 1e). The prevalence of elevated fasting glucose overall was 42.2% (95% CI: 40.0–44.4) and was higher in males than females (48.4% vs. 39.5%, *p* < 0.001) (Figure 1f) (Supplementary Table S1).

### 3.3. Association Analyses of Genetic Variants rs17120425 and rs1784042 of SIDT2 with MetS

In the first stage (pilot GWAS), none of the SNPs met the conventional criteria for a genome-wide significance (*p* < 10 × 10^−8^). There were not significant differences observed between MAF of the SNPs, compared to European populations (Supplementary Table S2). Therefore, we focused on the search for SNPs located in regions previously associated with MetS, TC, LDL-c, HDL-c and TG levels in European and Asian populations. In addition, we also considered the earlier know gene regions for MetS and related metabolic traits that were identified using NHGRI-EBI GWAS Catalog [33] and a PubMed search (Supplementary Table S3). The variant rs1784042, which is located within the *SIDT2* gene on chromosome 11, was the strongest signal (*p*_GWAS_ = 0.006) for HDL-c (Supplementary Figure S1 and Supplementary Table S4). The variant rs1784042 (A allele) has been recently associated with risk of MetS in a Korean population [16] and with high TG levels in cohorts from Nigeria and the Philippines [15], suggesting a potential association with MetS and related components in certain ethnic groups. In addition, due to the proximity to the variant rs1784042, we also included the missense variant p.V636I (rs17120425) and because, according to the 1000 genomes project, the rs17120425 is rare in several populations (MAF < 1.0%).

Supplementary Table S5 shows the MAF distribution of both polymorphisms of the *SIDT2* gene in different populations. The rs17120425 “A” allele was more frequent in the HWCS population (10%) than in the MXL population (6%). We observed that the rs17120425 “A” allele was rare or absent in the samples from Europe, South Asia and Africa. The SNP rs1784042 “A” allele showed a MAF similar to that reported in MXL sample (29% vs. 28%). This allele is rare (2%) in the African (YRI) samples and had an intermediate frequency (19%) in the Asian (CHB) samples, whereas a frequency of 46% was observed in the Northern/Western European population (CEU). Both SNPs were in HWE (*p* > 0.05).

The SNP rs17120425 was associated with MetS, only in the group of men under the additive model (OR = 0.60, *p* = 0.018) (Table 2). Regarding to the components of MetS, only low HDL-c levels showed association in the overall population (OR = 0.60, *p* = 4.3 × 10^−6^) and in women (OR = 0.57, *p* = 2 × 10^−5^), under the additive model. Additionally, the association was stronger in postmenopausal women (OR = 0.52, *p* = 0.0004) compared to premenopausal (Supplementary Table S6). Interestingly, in the overall population, we observed a significant association with T2D risk (OR = 1.39, *p* = 0.033). Females had a significantly higher risk of T2D than males (OR = 1.62, *p* = 0.007) (Table 2). These findings are supported by the quantitative analysis, this confirmed the association of the rs17120425 with HDL-c levels in all groups (Supplementary Table S7). We found no significant association with other metabolic components of MetS.

On the other hand, the SNP rs1784042 showed association in the overall population with MetS (OR = 0.82, *p* = 0.010). When the sample was stratified by gender, only women showed association with MetS, under the additive model (OR = 0.81, *p* = 0.020). Regarding the association with MetS components, only low HDL-c levels showed association in the overall population (OR = 0.77, *p* = 0.001) and in women (OR = 0.74, *p* = 0.001), under the additive model. No significant association with other components of MetS was observed (Table 3). The association between the SNP rs1784042 and low HDL-c levels was confirmed among women (Supplementary Table S8). However, the association was stronger in premenopausal women (OR = 0.68, *p* = 0.004), than in postmenopausal women (OR = 0.79, *p* = 0.048). The quantitative analysis confirmed the association between rs1784042 and HDL-c levels (β = 1.44, *p* = 0.0002) and TG levels (β = −13.35, *p* = 0.001) in all the population. Interestingly, this variant showed an increase in LDL-c levels in the total population (β = 2.51, *p* = 0.043) and a decrease in WC (β = −1.01, *p* = 0.011) (Supplementary Table S9).

We also analyzed the genotype distribution for each variant within the MetS components. The GA/AA genotypes of rs17120245 were significantly associated with higher HDL-c levels (*p* < 0.001 and *p* = 0.029, respectively) (Supplementary Figure S2a). Regarding rs1784042, we also observed that the HDL-c level was higher in carriers of the GA and AA genotype vs. GG (*p* = 0.023 and *p* = 0.0002, respectively) ( Supplementary Figure S2b). There were no significant differences between the genotype distribution and other metabolic component of MetS (data not shown).

The prevalence of low HDL-c was lower among carriers of the heterozygous genotype of rs17120425 and rs1784042 (47.4%, *p* < 0.001 and 57.7%, *p* = 0.013, respectively), than in individuals with the GG genotype (63.1% and 63.5%, respectively). Interestingly, only the prevalence of low HDL-c was lower in the AA genotype of the rs1784042 vs. GG genotype carriers (63.5% vs. 51.3%, *p* = 0.004) (Supplementary Figure S3b). In addition, we also observed a decrease in the prevalence of elevated WC in GA genotypes compared to GG carriers for both SNPs (*p* = 0.038 and *p* = 0.005, respectively) (Supplementary Figure S4). However, carriers of the AA genotype of rs1784042 had a minor prevalence of elevated WC compared to carriers of the GG genotype (*p* = 0.007) (Supplementary Figure S4b).

### 3.4. Conditional Analysis of SIDT2 Locus

In the conditional analysis, we observed that the association between the variants of *SIDT2* with MetS were not independent, in this study. However, the association of rs17120425 with low HDL-c was maintained upon adjusting for the rs1784042 variant (Supplemental Tables S10 and S11). The two SNPs are not in linkage disequilibrium (LD) with each other in the HWCS population (r^2^ = 0.18, D’ = 0.82) (Supplemental Figure S5a,b, respectively).

### 3.5. Haplotype Association Analysis

Haplotype analysis revealed that the haplotype AA (rs17120425-rs1784042) is strongly associated with low HDL-c in the HWCS population (OR = 0.52, *p* = 2.9 × 10^−8^), however, we did not observe association for MetS (Supplementary Table S12).

### 3.6. In Silico Functional Analysis of Genetic Variants rs17120425 and rs1784042 in SIDT2

In order to analyze the link between the SNPs (rs1784042 and rs17120425) in the *SIDT2* gene and gene expression, we used the online resources: the Genotype-Tissue Expression (GTEx) project (https://www.gtexportal.org/home/), the Netherlands Study of Depression and Anxiety (NESDA) and the Netherlands Twin Registry (NTR) Conditional Expression Quantitative Trait Loci (eQTL) catalog (https://eqtl.onderzoek.io/) and the RegulomeDB (http://www.regulomedb.org/). All three databases showed that the SNP rs1784042 correlated with *SIDT2* and *TAGLN* expression. The NESDA NTR catalog shows that rs1784042 correlates with *SIDT2, TAGLN* and *PAFAH1B2* expression, in whole blood. On the other hand, the analysis with the GTEx project showed that rs1784042 correlates with *SIDT2, TAGLN*, and *PCSK7* expression in whole blood (*p* = 2 × 10^−5^, *p* = 5.6 × 10^−18^ and *p* = 4.5 × 10^−5^, respectively), while the RegulomeDB database showed correlation between rs1784042 and the expression of *SIDT2* and *TAGLN* in monocytes.

No functional analysis was available for the V636I (rs17120425) variant in the online resources used. However, recently the Ile636 allele has been associated with high levels of HDL-c and has shown increased uptake of the cholesterol analog dehidroergosterol, in vitro [36].

## 4. Discussion

Our data extend the available information regarding the rs1784042 variant of the *SIDT2* gene, which confirms the association with MetS in the Mexican population. In addition, for the first-time, relevant information is provided about the association with HDL-c levels. To the best of our knowledge, this is the first study to suggest that the rs17120245 variant, regardless of rs1784042 genotype, increases T2D risk through lowering HDL-c plasma levels. The pairwise linkage disequilibrium score between rs17120425 and rs1784042 was low (r^2^ = 0.18), indicating little correlation between these two SNPs.

Interestingly, the presence of the SNP rs17120425 is observed almost exclusively in the American continent, with a MAF of 6%, unlike other populations around the world where it is rare or absent. Unlike rs1784042, which is present in all populations (MAF: 4–42%). Similar to the *ABCA1* gene [27] reported in the Mexican population, the ethnic specific effect might result from the well-recognized variation of the *SIDT2* alleles that exists between populations of different origins. These alleles may be preserved in the Native American populations because it provided some adaptive advantage in the past [37,38].

These data also provide information regarding the association between the missense variant Val636Ile (rs17120425) on the *SIDT2* gene and HDL-c levels. In addition, we found that effect of both variants on the HDL-c levels appears to be greater in women. Although the reason for the gender specific differences remains unclear, gender specific effects is commonly observed in complex traits [39]. Our findings are in line with previous studies [40] and it has been observed that the prevalence of MetS increases with age in females, very similar to what was observed in this study. Moreover, females are more susceptible to MetS due to many cultural factors including stress and low socioeconomic status [41].

Our results suggest that the rs17120245 variant, regardless of rs1784042 genotype, increases T2D risk through lowering HDL-c levels. This is the first report revealing HDL-c concentrations as an intermediate factor between a *SIDT2* gene variant and T2D risk. This finding is consistent with previous studies that show that the function of *SIDT2* is related to dysfunction on glucose metabolism, which was manifested as increased random blood glucose level and impaired glucose tolerance [22]. This is in line with previous epidemiological studies that have identified HDL-c levels as a risk factor for T2D [42,43]. Some in vitro, animal and clinical studies have uncovered a broad range of HDL-c actions contributing to the pathophysiology of T2D, and therefore various mechanisms have been proposed [44,45,46,47,48,49]. Diverse studies suggest that reduced ATP-binding cassette transporter A1 (ABCA1) activity leads to impaired β-cell function [44,46]. HDL-c may raise insulin secretion through an increase in cholesterol efflux [45]. Studies aimed to document the complexity of the etiology and the influence of gender, age, and environmental factors should be investigated, in the Mexican population.

First insights into the roles of *SIDT2* were derived from studies of *Sidt2* knockout mice [22,23] that showed elevated fasting glucose levels, glucose intolerance and decreased serum insulin levels. Other studies have identified a role of *Sidt2* also in lipid metabolism. *Sidt2*-deficient mice developed an increase in serum of TG, FFAs and liver transaminases, indicating an impaired of liver function [16,19,22]. Recently, Mendez-Acevedo et al. suggested that *SIDT2* is involved in cholesterol transport, but not in RNA transport [21]. However, more studies are needed to elucidate the function of this gene and its impact on MetS and its mainly component HDL-c in the Mexican population. The Mexican population has one of the highest rates of low HDL-c levels and prevalences of MetS worldwide [3,50,51].

Our findings are supported by previous studies that indicate the role of the rs1784042 variant in lipid metabolism. Gombojav et al. identified in more than 8000 Korean individuals, several functional loci in 11q23.3 and found that *SIDT2* is associated with an effect on plasma TG levels [14]. These data are consistent with the findings from Moon et al., who carried out a multiple genotype-phenotype association study in more than 10,000 Korean subjects and identified that the variant rs1784042 was associated with MetS and its components [16,52]. In addition, Kulminsky et al. in a GWAS with more than 26,000 individuals from five longitudinal studies identified that the rs1784042 variant was associated with TC levels [17].

Along with previous reports, our study supports the hypothesis that genes are key factors in the link between lipid metabolism and MetS [11,53]. We suggest that lipid traits such as HDL-c have the greatest impact on MetS, but other weak associations, such as TG levels and WC, may also have an impact on the Mexican population. Interestingly, our study suggests that the genetic variant rs1784042 could have pleiotropic effects for MetS, in Asian populations it is correlated with TG levels, while in Mexicans it is associated mainly with HDL-c levels. However, due to the limitations of the current research, the potential weak association must be interpreted carefully.

Data derived from the three online databases showed that rs1784042 correlates with the expression of *SIDT2* and *TAGLN*. We assume that the SNPs associated with MetS could activate the expression of the genes *SIDT2* and *TAGLN*. A change in gene expression might inhibit insulin secretion, lipid metabolism and adipogenesis, resulting in MetS. Whereas for V636I (rs17120425), recently the Ile636 allele has been associated with increased cholesterol analog uptake in vitro [36]. Although evidence supports the association between the SNPs rs1784042 and rs17120425 with HDL-c levels, the mechanism affecting HDL-c levels in MetS is unknown. Additional functional investigations for both SNPs are needed. Although previously identified variants are in lipid loci, these variants and their mapped genes are not reported in MetS cases. Our findings provide insight into the genetic variant contribution to MetS risk, in Mexicans.

The information about the prevalence of MetS and its components among the Mexican population is limited and data on uniform MetS diagnostic criteria are even more scarce, as are reasons why it hinders the initiatives to prevent metabolic disorders in this population. The HWCS study showed an overall prevalence 52.6% of MetS, the main findings were low HDL-c levels and elevated WC. The MetS prevalence found in this study was higher than that reported in other studies of health-care workers (29.5–40%) [3,54,55,56,57,58,59,60,61,62]. However, it was similar to the prevalence observed in the general population of Mexican-Amerindians (50.3%) [50]. Discrepancies between the overall prevalence of MetS in this study and in the above-mentioned studies may be attributable to the fact that most of the subjects in this study were older than 40 years old and presented high obesity rates. As found in other published studies, MetS frequency increase with age [3,56,63] and BMI [64,65]. Thereby, it is evident that the prevalence of MetS is increasing in the general population.

The MetS component most altered was low levels of HDL-c followed by elevated WC. Low levels of HDL-c are common in the Mexican population, which has one of the highest rates worldwide [51]. Previous reports confirm that elevated WC and low HDL-c levels are the most frequently components of MetS on general population [3,64]. This may be attributable to Latin American populations which are more susceptible to accumulation of abdominal fat, the development of insulin resistance and fatty liver than non-Hispanic white populations [66].

This is the first candidate gene association study showing the role of genetic variants of the *SIDT2* gene in MetS and low HDL-c levels in the Mexican population. The sample size (*n* = 1963) was large enough and robustly powered to detect associations with similar ORs as identified in reported GWAS and candidate-gene studies for MetS. The statistical power of this study was >90% to detect previous associations observed in the literature.

Although we observed statistically significant associations, our study has some potential limitations. First, this cohort contained adults from a specific segment of the Mexican population, mainly composed of health workers and their relatives, who live in central Mexico. While these adults cannot be considered representative of the Mexican adult population as a whole, they may be considered representative of the middle-income sector, which may limit the generalizability of our results to the general population. Second, the lack of information about other genetic variants (i.e., *ABCA1* genotypes) or environmental factors that may affect HDL-c concentrations is another limitation. Even in light of these limitations, our results provide important knowledge about the association between *SIDT2* with MetS and low levels of HDL-c, in the health professionals population.

The strengths of our study include associations that were adjusted for variables accounted for in previous studies (e.g., age, sex), which allow consistency and comparability of the results. However, this did not have an effect on the estimators of this study. The GWAS analyses in the discovery sample (22% of the total of the sample) were adjusted for ancestry to reduce the type 2 error that could be caused by stratification of the population.

## 5. Conclusions

In conclusion, our study suggests a protective effect of rs1784042 and rs17120425 *SIDT2* gene variants for low levels of HDL-c. Furthermore, rs1784042 *SIDT2* gene variant showed association with MetS and rs17120425 variant suggested an association with T2D. The current findings provide preliminary insights into the role of these SNPs in the manifestation of MetS. However, further studies are required to confirm these associations. Moreover, additional functional studies, both in vitro and in vivo, are required to further understand the role of *SIDT2* and the V676I (rs17120425) and rs1784042 variants in HDL-c levels.

## Figures and Tables

**Table 1:** Sociodemographic and clinical characteristics of the Health Workers Cohort Study (HWCS).

Parameter	Total Sample	Men	Women	*p* Value
	*n* = 1963	*n* = 593	*n* = 1370	
Age(years) ^1^	52.0 (40.0–62.0)	46.0 (36.0–57.0)	54.0 (43.0–63.0)	<0.001
Body mass index (kg/m^2^) ^1^	26.7 (24.0–29.7)	26.5 (24.1–29.0)	26.8 (24.0–30.1)	0.133
Overweight ^2^	42.9 (40.7–45.0)	48.8 (44.9–52.8)	40.2 (37.7–42.8)	<0.001
Obesity ^2^	23.9 (22.0–25.8)	19.6 (16.5–22.8)	25.7 (23.4–28.0)	0.003
Waist circumference (cm) ^1^	94.0 (86.0–101.0)	96.0 (90.0–102.0)	92.0 (85–100.0)	<0.001
Body fat proportion ^1^	41.9 (34.0–47.2)	31.5 (27.7–34.7)	45.1 (40.7–49.1)	<0.001
Leisure time physical activity (hour/week) ^1^	1.5 (0.3–3.5)	1.7 (0.4–5.0)	1.1 (0.2–3.5)	<0.001
Active (>150 min/week) ^2^	34.9 (32.8–37.0)	36.6 (32.8–40.4)	28.2 (25.8–30.6)	<0.001
Smoking current ^2^	12.3 (10.9–13.7)	21.3 (18.0–26.6)	9.0 (7.5–10.6)	<0.001
Smoking past ^2^	27.5 (25.5–29.4)	39.7 (35.7–43.6)	23.8 (21.5–26.1)	<0.001
Systolic blood pressure (mmHg) ^1^	118.0 (108.0–129.5)	122.0 (113.0–131.0)	116.0 (106.0–129.0)	<0.001
Diastolic blood pressure (mmHg) ^1^	74.0 (68.0–81.0)	77.0 (70–84)	73.0 (66.0–79.0)	<0.001
Fasting plasma glucose (mg/dL) ^1^	97.0 (90.0–106.0)	98.0 (92–107)	96.0 (90.0–104.0)	<0.001
Total cholesterol (mg/dL) ^1^	140.6 (85.4–213.6)	167.0 (105.0–266.0)	128.0 (83.0–193.0)	<0.001
Low density lipoprotein-c (mg/dL) ^1,3^	120.0 (98.0–145.4)	116.0 (97–143.0)	121.0 (99.0–147.0)	0.009
High density lipoprotein-c (mg/dL) ^1,4^	44.0 (37.0–52.0)	39.0 (34.0–46.0)	46.0 (39.0–54.0)	<0.001
Metabolic Syndrome ^2,5^	52.6 (50.4–54.8)	45.7 (41.6–49.7)	55.6 (52.9–58.3)	<0.001
Triglycerides (mg/dL) ^1^	156.0 (112.0–209.0)	168.0 (118.0–247.0)	151.0 (109.0–199.0)	<0.001

^1^ Median(P25-P75). ^2^ Percent (95% Confidence Interval). ^3^ LDL-c. ^4^ HDL-c. ^5^ MetS, (Adult Treatment Panel (ATP)-III definition).

**Table 2:** Association of rs17120425 polymorphism and metabolic syndrome.

		Total				Men				Women			
Outcome	Genotype	Control, *n* (%)	Case, *n* (%)	OR ^1,7^ (95% CI)	*p* Value	Control, *n* (%)	Case, *n* (%)	OR ^2^ (95% CI)	*p* Value	Control, *n* (%)	Case, *n* (%)	OR ^2^ (95% CI)	*p* Value
MetS ^3^	GG	742 (80.2)	858 (83.3)	Ref.		256 (80.3)	232 (86.3)	Ref.		486 (80.2)	626 (82.4)	Ref.	
	GA	173 (18.8)	160(15.5)	0.79 (0.61–1.01)	0.065	58 (18.2)	36 (13.4)	0.66 (0.41–1.05)	0.078	115 (19.0)	124 (16.3)	0.86 (0.63–1.15)	0.307
	AA	10 (1.0)	11 (1.1)	0.83 (0.33–2.10)	0.700	5 (1.6)	1 (0.4)	0.15 (0.02–1.39)	0.095	5 (0.8)	10 (1.3)	1.59 (0.48–5.35)	0.450
	Additive model			0.82 (0.65–1.02)	0.074			0.60 (0.39–0.92)	0.018			0.93 (0.71–1.21)	0.575
Low HDL- cholesterol ^4^	GG	514 (76.4)	1086 (84.8)	Ref.		203 (79.9)	285 (85.3)	Ref.		311 (74.2)	801 (84.6)	Ref.	
	GA	151 (22.4)	182 (14.2)	0.54 (0.42–0.69)	7.5 × 10^−7^	48 (18.9)	46 (13.8)	0.65 (0.42–1.02)	0.059	103 (24.6)	136 (14.4)	0.50 (0.37–0.66)	2.7 × 10^−6^
	AA	8 (1.2)	13 (1.0)	0.73 (0.30–1.79)	0.492	3 (1.2)	3 (0.9)	0.66 (0.13–3.32)	0.613	5 (1.2)	10 (1.1)	0.78 (0.26–2.31)	0.652
	Additive model			0.60 (0.48–0.74)	4.3 × 10^−6^			0.68 (0.46–1.02)	0.062			0.57 (0.44–0.74)	2 × 10^−5^
Impaired glucose tolerance ^5^	GG	934 (82.6)	446 (82.8)	Ref.		251 (82.0)	165 (84.6)	Ref.		683 (82.8)	281 (81.7)	Ref.	
	GA	184 (16.3)	89 (16.5)	1.03 (0.77–1.38)	0.825	50 (16.3)	30 (15.4)	0.84 (0.51–1.40)	0.51	134 (16.2)	59 (17.2)	1.12 (0.79–1.59)	0.52
	AA	13 (1.2)	4 (0.7)	0.54 (0.17–1.70)	0.292	5 (1.6)	-			8 (1.0)	4 (1.2)	1.01 (0.30–3.47)	0.983
	Additive model			0.96 (0.74–1.25)	0.773			0.70 (0.44–1.12)	0.135			1.10 (0.80–1.50)	0.56
Type 2 Diabetes ^6^	GG	934 (82.6)	220 (77.6)	Ref.		251 (82.0)	72 (82.8)	Ref.		683 (82.8)	148 (75.1)	Ref.	
	GA	184 (16.3)	60 (21.1)	1.56 (1.11–2.21)	0.011	50 (16.3)	14 (16.1)	0.98 (0.49–1.97)	0.954	134 (16.2)	46 (23.4)	1.82 (1.22–2.71)	0.003
	AA	13 (1.2)	4 (1.4)	0.97 (0.29–3.20)	0.959	5 (1.6)	1 (1.2)	0.32 (0.03–3.61)	0.355	8 (1.0)	3 (1.5)	1.39 (0.35–5.54)	0.644
	Additive model			1.39 (1.03–1.88)	0.033			0.86 (0.46–1.59)	0.634			1.62 (1.14–2.30)	0.007

^1^ Model adjusted for age, sex and family group. ^2^ Model adjusted for age and family group. ^3^ MetS: Metabolic Syndrome (ATP-III definition). ^4^ Low HDL-cholesterol: <40 in men and <50 in women. ^5^ Impaired glucose tolerance: >100 to <126 glucose levels. ^6^ Type 2 diabetes: >126 glucose levels or self-report of physician diagnosis. ^7^ OR: Odd Ratio.

**Table 3:** Association of rs1784042 polymorphism and metabolic syndrome.

		Total				Men				Women			
Outcome	Genotype	Control, *n* (%)	Case, *n* (%)	OR ^1,7^ (95% CI)	*p* Value	Control, *n* (%)	Case, *n* (%)	OR ^2^ (95% CI)	*p* Value	Control, *n* (%)	Case, *n* (%)	OR ^2^ (95% CI)	*p* Value
MetS ^3^	GG	445 (49.0)	534 (52.0)	Ref.		161 (50.2)	138 (51.3)	Ref.		294 (48.4)	396 (52.2)	Ref.	
	GA	391 (42.1)	417 (40.6)	0.84 (0.69–1.03)	0.097	135 (42.1)	117 (43.5)	0.98 (0.67–1.34)	0.780	256 (42.2)	300 (39.5)	0.80 (0.62–1.02)	0.066
	AA	82 (8.8)	77 (7.5)	0.65 (0.45–0.93)	0.018	25 (7.8)	14 (5.2)	0.59 (0.29–1.19)	0.138	63 (8.3)	63 (8.3)	0.67 (0.44–1.02)	0.061
	Additive model			0.82 (0.71–0.95)	0.010			0.85 (0.65–1.12)	0.258			0.81 (0.68–0.97)	0.020
Low HDL-cholesterol ^4^	GG	308 (45.5)	681 (53.2)	Ref.		124 (48.4)	175 (52.4)	Ref.		184 (43.7)	506 (53.5)	Ref.	
	GA	304 (44.9)	504 (39.4)	0.75 (0.61, 0.91)	0.004	112 (43.8)	140 (41.9)	0.90 (0.64–1.27)	0.546	192 (45.6)	364 (38.5)	0.67 (0.53–0.86)	0.002
	AA	65 (9.6)	94 (7.4)	0.63 (0.45, 0.90)	0.011	20 (7.8)	19 (5.7)	0.66 (0.34–1.30)	0.23	45 (10.7)	75 (7.9)	0.61 (0.40–0.93)	0.020
	Additive model			0.77 (0.67, 0.90)	0.001			0.85 (0.65–1.12)	0.249			0.74 (0.62–0.88)	0.001
Impaired glucose tolerance ^5^	GG	567 (50.3)	280 (51.7)	Ref.		158 (52.0)	102 (51.5)	Ref.		409 (49.6)	178 (51.7)	Ref.	
	GA	468 (41.5)	222 (41.0)	0.93 (0.75, 1.16)	0.542	126 (41.5)	87 (43.9)	1.04 (0.71–1.52)	0.831	342 (41.5)	135 (39.2)	0.89 (0.69–1.17)	0.418
	AA	93 (8.2)	40 (7.4)	0.83 (0.55, 1.26)	0.388	20 (6.6)	9 (4.6)	0.65 (0.28–1.50)	0.309	73 (8.9)	31 (9.0)	0.92 (0.57–1.47)	0.729
	Additive model			0.92 (0.78, 1.09)	0.344			0.93 (0.69–1.26)	0.625			0.93 (0.76–1.14)	0.492
Type 2 Diabetes ^6^	GG	567 (50.3)	142 (49.7)	Ref.		158 (52.0)	39 (44.3)	Ref.		409 (49.6)	103 (52.0)	Ref.	
	GA	468 (41.5)	118 (41.3)	0.96 (0.72, 1.29)	0.800	126 (41.5)	39 (44.3)	1.25 (0.72–2.16)	0.43	342 (41.5)	79 (39.9)	0.87 (0.62–1.23)	0.432
	AA	93 (8.2)	26 (9.1)	0.99 (0.60, 1.63)	0.967	20 (6.6)	10 (11.4)	1.76 (0.70–4.41)	0.23	73 (8.9)	16 (8.1)	0.78 (0.42–1.43)	0.419
	Additive model			0.98 (0.79, 1.22)	0.87			1.31 (0.88–1.97)	0.187			0.88 (0.68–1.13)	0.314

^1^ Model adjusted for age, sex and family group. ^2^ Model adjusted for age and family group. ^3^ MetS: Metabolic Syndrome (ATP-III definition). ^4^ Low HDL-cholesterol: <40 in men and <50 in women; ^5^ Impaired glucose tolerance: >100 to <126 glucose levels; ^6^ Type 2 diabetes: >126 glucose levels or self-report of physician diagnosis. ^7^ OR: Odd Ratio.

## Supplementary Materials

The following are available online at https://www.mdpi.com/2073-4425/11/10/1192/s1 Table S1: Prevalence of MetS and its components by gender and age categories, Table S2: Top SNPs associated with total cholesterol, HDLc, LDL-c and triglycerides in the European and discovery population (pilot HWCS), Table S3: Earlier known gene regions for MetS and related metabolic traits, Table S4: Genetic region on chromosome 11 associated with HDL-c in pilot Genome-wide association study (GWAS) of BMD and metabolic traits, Table S5: Allele frequencies of *SIDT2* gene polymorphisms, Table S6: Association of rs17120425 polymorphism and MetS by postmenopausal status, Table S7: Association between rs17120425 and HDL-c levels, Table S8: Association of rs1784042 polymorphism and MetS by postmenopausal status, Table S9: Association between rs1784042 and components of the MetS, Table S10: Conditional analysis for rs17120425 and MetS and its components in additive models, Table S11: Conditional analysis for rs1784042 and MetS and its components in additive models, Table S12: Haplotype Association Analysis of *SIDT2* gene SNPs and MetS and its components, Figure S1: LocusZoom plot showing the HDL-c associated region, Figure S2: HDL-c levels by genotypes of *SIDT2* gene variants, Figure S3: Prevalence of low HDL-c by genotypes of *SIDT2* gene variants, Figure S4: Prevalence of high waist circumference by genotypes of *SIDT2* gene variants, Figure S5: Pairwise linkage disequilibrium (LD) between the two *SIDT2* variants associated with MetS and HDL-c, in the present study.

## References

[1] McNeill A.M., Rosamond W.D., Girman C.J., Golden S.H., Schmidt M.I., East H.E., Ballantyne C.M., Heiss G. (2005). The metabolic syndrome and 11-year risk of incident cardiovascular disease in the atherosclerosis risk in communities study. Diabetes Care.

[2] Punthakee Z., Goldenberg R., Katz P. (2018). 2018 Clinical Practice Guidelines Definition, Classification and Diagnosis of Diabetes, Prediabetes and Metabolic Syndrome Diabetes Canada Clinical Practice Guidelines Expert Committee. Can. J. Diabetes.

[3] Rojas R., Aguilar-Salinas C.A., Jiménez-Corona A., Shamah-Levy T., Rauda J., Ávila-Burgos L., Villalpando S., Lazcano Ponce E. (2010). Metabolic syndrome in Mexican adults: Results from the National Health and Nutrition Survey 2006. Salud Publica Mex..

[4] Terán-García M., Bouchard C. (2007). Genetics of the metabolic syndrome. Appl. Physiol. Nutr. Metab..

[5] Watanabe R.M., Valle T., Hauser E.R., Ghosh S., Eriksson J., Kohtamäki K., Ehnholm C., Tuomilehto J., Collins F.S., Bergman R.N. (1999). Familiality of quantitative metabolic traits in Finnish families with non-insulin-dependent diabetes mellitus. Finland-United States Investigation of NIDDM Genetics (FUSION) Study investigators. Hum. Hered..

[6] Ziki M.D.A., Mani A. (2016). Metabolic syndrome: Genetic insights into disease pathogenesis. Curr. Opin. Lipidol..

[7] Willer C.J., Schmidt E.M., Sengupta S., Peloso G.M., Gustafsson S., Kanoni S., Ganna A., Chen J., Buchkovich M.L., Mora S. (2013). Discovery and refinement of loci associated with lipid levels. Nat. Genet..

[8] Bandesh K., Prasad G., Giri A.K., Kauser Y., Upadhyay M., Basu A., Tandon N., Bharadwaj D. (2019). Genome-wide association study of blood lipids in Indians confirms universality of established variants. J. Hum. Genet..

[9] Dupuis J., Langenberg C., Prokopenko I., Saxena R., Soranzo N., Jackson A.U., Wheeler E., Glazer N.L., Bouatia-Naji N., Gloyn A.L. (2010). New genetic loci implicated in fasting glucose homeostasis and their impact on type 2 diabetes risk. Nat. Genet..

[10] Stančáková A., Laakso M. (2014). Genetics of metabolic syndrome. Rev. Endocr. Metab. Disord..

[11] Kristiansson K., Perola M., Tikkanen E., Kettunen J., Surakka I., Havulinna A.S., Stančáková A., Barnes C., Widen E., Kajantie E. (2012). Genome-wide screen for metabolic syndrome susceptibility loci reveals strong lipid gene contribution but no evidence for common genetic basis for clustering of metabolic syndrome traits. Circ. Cardiovasc. Genet..

[12] Zafar U., Khaliq S., Ahmad H.U., Manzoor S., Lone K.P. (2018). Metabolic syndrome: An update on diagnostic criteria, pathogenesis, and genetic links. Hormones.

[13] Brown B.C., Ye C.J., Price A.L., Zaitlen N. (2016). Transethnic Genetic-Correlation Estimates from Summary Statistics. Am. J. Hum. Genet..

[14] Gombojav B., Lee S.J., Kho M., Song Y.M., Lee K., Sung J. (2016). Multiple susceptibility loci at chromosome 11q23.3 are associated with plasma triglyceride in East Asians. J. Lipid Res..

[15] Andaleon A., Mogil L.S., Wheeler H.E. (2018). Gene-based association study for lipid traits in diverse cohorts implicates BACE1 and SIDT2 regulation in triglyceride levels. PeerJ.

[16] Moon S., Lee Y., Won S., Lee J. (2018). Multiple genotype-phenotype association study reveals intronic variant pair on SIDT2 associated with metabolic syndrome in a Korean population. Hum. Genomics.

[17] Kulminski A.M., Loika Y., Huang J., Arbeev K.G., Bagley O., Ukraintseva S., Yashin A.I., Culminskaya I. (2019). Pleiotropic meta-analysis of age-related phenotypes addressing evolutionary uncertainty in their molecular mechanisms. Front. Genet..

[18] Gao J., Zhang Y., Yu C., Tan F., Wang L. (2016). Spontaneous nonalcoholic fatty liver disease and ER stress in Sidt2 deficiency mice. Biochem. Biophys. Res. Commun..

[19] Chen X., Gu X., Zhang H. (2018). Sidt2 regulates hepatocellular lipid metabolism through autophagy. J. Lipid Res..

[20] Jialin G., Xuefan G., Huiwen Z. (2010). SID1 transmembrane family, member 2 (Sidt2): A novel lysosomal membrane protein. Biochem. Biophys. Res. Commun..

[21] Méndez-Acevedo K.M., Valdes V.J., Asanov A., Vaca L. (2017). A novel family of mammalian transmembrane proteins involved in cholesterol transport. Sci. Rep..

[22] Gao J., Gu X., Mahuran D.J., Wang Z., Zhang H. (2013). Impaired Glucose Tolerance in a Mouse Model of Sidt2 Deficiency. PLoS ONE.

[23] Gao J., Yu C., Xiong Q., Zhang Y., Wang L. (2015). Lysosomal integral membrane protein Sidt2 plays a vital role in insulin secretion. Int. J. Clin. Exp. Pathol..

[24] Moreno-Estrada A., Gignoux C.R., Fernández-López J.C., Zakharia F., Sikora M., Contreras A.V., Acuña-Alonzo V., Sandoval K., Eng C., Romero-Hidalgo S. (2014). The genetics of Mexico recapitulates Native American substructure and affects biomedical traits. Science.

[25] Pedroza-Tobias A., Trejo-Valdivia B., Sanchez-Romero L.M., Barquera S. (2014). Classification of metabolic syndrome according to lipid alterations: Analysis from the Mexican National Health and Nutrition Survey 2006. BMC Public Health.

[26] Aguilar-Salinas C.A., Olaiz G., Valles V., Torres J.M.R., Gómez Pérez F.J., Rull J.A., Rojas R., Franco A., Sepulveda J. (2001). High prevalence of low HDL cholesterol concentrations and mixed hyperlipidemia in a Mexican nationwide survey. J. Lipid Res..

[27] Acuña-Alonzo V., Flores-Dorantes T., Kruit J.K., Villarreal-Molina T., Arellano-Campos O., Hünemeier T., Moreno-Estrada A., Ortiz-López M.G., Villamil-Ramírez H., León-Mimila P. (2010). A functional ABCA1 gene variant is associated with low HDL-cholesterol levels and shows evidence of positive selection in Native Americans. Hum. Mol. Genet..

[28] Williams A.L., Jacobs S.B.R., Moreno-Macías H., Huerta-Chagoya A., Churchhouse C., Márquez-Luna C., García-Ortíz H., Gómez-Vázquez M.J., The SIGMA Type 2 Diabetes Consortium (2014). Sequence variants in SLC16A11 are a common risk factor for type 2 diabetes in Mexico. Nature.

[29] Villalobos-Comparán M., Jiménez-Ortega R.F., Estrada K., Parra-Torres A.Y., González-Mercado A., Patiño N., Castillejos-López M., Quiterio M., Fernandez-López J.C., Ibarra B. (2017). A pilot genome-wide association study in postmenopausal Mexican-Mestizo women implicates the RMND1/CCDC170 locus is associated with bone mineral density. Int. J. Genomics.

[30] Rivera-Paredez B., Macías-Kauffer L., Fernandez-Lopez J.C., Villalobos-Comparán M., Martinez-Aguilar M.M., De la Cruz-Montoya A., Ramírez-Salazar E.G., Villamil-Ramírez H., Quiterio M., Ramírez-Palacios P. (2019). Influence of genetic and non-genetic risk factors for serum uric acid levels and hyperuricemia in mexicans. Nutrients.

[31] Denova-Gutierrez E., Flores Y.N., Gallegos-Carrillo K., Ramirez-Palacios P., Rivera-Paredez B., Munoz-Aguirre P., Velazquez-Cruz R., Torres-Ibarra L., Meneses-Leon J., Mendez-Hernandez P. (2016). Health workers cohort study: Methods and study design. Salud Publica Mex..

[32] Loh P.R., Danecek P., Palamara P.F., Fuchsberger C., Reshef Y.A., Finucane H.K., Schoenherr S., Forer L., McCarthy S., Abecasis G.R. (2016). Reference-based phasing using the Haplotype Reference Consortium panel. Nat. Genet..

[33] MacArthur J., Bowler E., Cerezo M., Gil L., Hall P., Hastings E., Junkins H., McMahon A., Milano A., Morales J. (2017). The new NHGRI-EBI Catalog of published genome-wide association studies (GWAS Catalog). Nucleic Acids Res..

[34] Gauderman W.J. (2002). Sample size requirements for association studies of gene-gene interaction. Am. J. Epidemiol..

[35] Barrett J.C., Fry B., Maller J., Daly M.J. (2005). Haploview: Analysis and visualization of LD and haplotype maps. Bioinformatics.

[36] Leon-Mimila P., Villamil-Ramirez H., Macias-Kauffer L.R., Jacobo-Albavera L., Lopez-Contreras B.E., Posadas-Sanchez R., Posadas-Romero C., Romero-Hidalgo S., Moran-Ramos S., Dominguez-Perez M. (2020). A functional variant of the SIDT2 gene involved in cholesterol transport is associated with HDL-C levels and premature coronary artery disease. medRxiv.

[37] Aguilar Salinas C., Cruz-Bautista I., Mehta R., Villarreal-Molina M., Perez F., Tusie-Luna M., Canizales-Quinteros S. (2007). The ATP-Binding Cassette Transporter Subfamily A Member 1 (ABC-A1) and Type 2 Diabetes: An Association Beyond HDL Cholesterol. Curr. Diabetes Rev..

[38] Aguilar-Salinas C.A., Canizales-Quinteros S., Rojas-Martfnez R., Mehta R., Ma T.V.M., Arellano-Campos O., Riba L., Gómez-Pérez F.J., Tusié-Luna M.T. (2009). Hypoalphalipoproteinemia in populations of Native American ancestry: An opportunity to assess the interaction of genes and the environment. Curr. Opin. Lipidol..

[39] Khramtsova E.A., Davis L.K., Stranger B.E. (2019). The role of sex in the genomics of human complex traits. Nat. Rev. Genet..

[40] Kong S., Cho Y.S. (2019). Identification of female-specific genetic variants for metabolic syndrome and its component traits to improve the prediction of metabolic syndrome in females. BMC Med. Genet..

[41] Pucci G., Alcidi R., Tap L., Battista F., Mattace-Raso F., Schillaci G. (2017). Sex- and gender-related prevalence, cardiovascular risk and therapeutic approach in metabolic syndrome: A review of the literature. Pharmacol. Res..

[42] Abbasi A., Corpeleijn E., Gansevoort R.T., Gans R.O.B., Hillege H.L., Stolk R.P., Navis G., Bakker S.J.L., Dullaart R.P.F. (2013). Role of HDL cholesterol and estimates of HDL particle composition in future development of type 2 diabetes in the general population: The PREVEND study. J. Clin. Endocrinol. Metab..

[43] Hirano M., Nakanishi S., Kubota M., Maeda S., Yoneda M., Yamane K., Kira S., Sasaki H., Kohno N. (2014). Low high-density lipoprotein cholesterol level is a significant risk factor for development of type 2 diabetes: Data from the Hawaii-Los Angeles-Hiroshima study. J. Diabetes Investig..

[44] Brunham L.R., Kruit J.K., Pape T.D., Timmins J.M., Reuwer A.Q., Vasanji Z., Marsh B.J., Rodrigues B., Johnson J.D., Parks J.S. (2007). β-cell ABCA1 influences insulin secretion, glucose homeostasis and response to thiazolidinedione treatment. Nat. Med..

[45] Brunham L.R., Kruit J.K., Verchere C.B., Hayden M.R. (2008). Cholesterol in islet dysfunction and type 2 diabetes. J. Clin. Investig..

[46] Vergeer M., Brunham L.R., Koetsveld J., Kruit J.K., Verchere C.B., Kastelein J.J.P., Hayden M.R., Stroes E.S.G. (2010). Carriers of loss-of-function mutations in ABCA1 display pancreatic β-cell dysfunction. Diabetes Care.

[47] Fryirs M.A., Barter P.J., Appavoo M., Tuch B.E., Tabet F., Heather A.K., Rye K.A. (2010). Effects of high-density lipoproteins on pancreatic β-cell insulin secretion. Arterioscler. Thromb. Vasc. Biol..

[48] Cochran B.J., Bisoendial R.J., Hou L., Glaros E.N., Rossy J., Thomas S.R., Barter P.J., Rye K.A. (2014). Apolipoprotein A-I increases insulin secretion and production from pancreatic β-cells via a G-protein-cAMPPKA-FoxO1-dependent mechanism. Arterioscler. Thromb. Vasc. Biol..

[49] Von Eckardstein A., Widmann C. (2014). High-density lipoprotein, β cells, and diabeteś. Cardiovasc. Res..

[50] Mendoza-Caamal E.C., Barajas-Olmos F., Garciá-Ortiz H., Cicerón-Arellano I., Martínez-Hernández A., Córdova E.J., Esparza-Aguilar M., Contreras-Cubas C., Centeno-Cruz F., Cid-Soto M. (2020). Metabolic syndrome in indigenous communities in Mexico: A descriptive and cross-sectional study. BMC Public Health.

[51] Aguilar-Salinas C.A., Canizales-Quinteros S., Rojas-Martínez R., Mehta R., Rodriguez-Guillén R., Ordoñez-Sanchez M.L., Riba L., Tusié-Luna M.T. (2011). The non-synonymous Arg230Cys variant (R230C) of the ATP-binding cassette transporter A1 is associated with low HDL cholesterol concentrations in Mexican adults: A population based nation wide study. Atherosclerosis.

[52] Veenstra J., Kalsbeek A., Koster K., Ryder N., Bos A., Huisman J., Vanderberg L., Vanderwoude J., Tintle N.L. (2018). Epigenome wide association study of SNP-CpG interactions on changes in triglyceride levels after pharmaceutical intervention: A GAW20 analysis 06 Biological Sciences 0604 Genetics. BMC Proc..

[53] Povel C.M., Boer J.M.A., Reiling E., Feskens E.J.M. (2011). Genetic variants and the metabolic syndrome: A systematic review. Obes. Rev..

[54] Orozco-González N., Cortés-Sanabria L., Viera-Franco J.J., Ramírez-Márquez J.J., Cueto-Manzano A.M. (2016). Prevalence of cardiovascular risk factors in a population of health-care workers. Rev. Med. Inst. Mex. Seguro Soc..

[55] Padierna-Luna J.L., Ochoa-Rosas F.S., Jaramillo-Villalobos B. (2007). Prevalence of metabolic syndrome in health employees. Rev. Med. Inst. Mex. Seguro Soc..

[56] Palacios-Rodríguez R.G., Paulín-Villalpando P., López-Carmona J.M., Valerio-Acosta M.M.L., Cabrera-Gaytán D.A. (2010). Metabolic syndrome in health care personnel from a primary care unit. Rev. Med. Inst. Mex. Seguro Soc..

[57] Mathiew-Quirós Á., Salinas-Martínez A.M., Hernández-Herrera R.J., Gallardo-Vela J.A. (2014). Metabolic syndrome in workers of a second level hospital. Rev. Med. Inst. Mex. Seguro Soc..

[58] Cruz-Dominguez M.P., González-Márquez F., Ayala-López E.A., Vera-Lastra L.O., Vargas- Rendón G.H., Zarate-Amador A., Jara-Quezada L.J. (2015). Sobrepeso, obesidad, síndrome metabólico e índice cintura/talla en el personal de salud [Overweight, obesity, metabolic syndrome and waist/height index in health staff]. Rev. Med. Inst. Mex. Seguro Soc..

[59] Lavalle F.J., Villarreal J.Z., Montes J., Mancillas L.G., Rodríguez S.E., González P., Lara R. (2015). Change in the prevalence of metabolic syndrome in a population of medical students: 6-year follow-up. J. Diabetes Metab. Disord..

[60] Vizmanos B., Betancourt-Nuñez A., Márquez-Sandoval F., González-Zapata L.I., Monsalve-Álvarez J., Bressan J., De Carvalho Vidigal F., Figueredo R., López L.B., Babio N. (2020). Metabolic Syndrome among Young Health Professionals in the Multicenter Latin America Metabolic Syndrome Study. Metab. Syndr. Relat. Disord..

[61] Moore J.X., Chaudhary N., Akinyemiju T. (2017). Metabolic syndrome prevalence by race/ethnicity and sex in the united states, national health and nutrition examination survey, 1988–2012. Prev. Chronic Dis..

[62] Márquez-Sandoval F., MacEdo-Ojeda G., Viramontes-Hörner D., Fernández Ballart J.D., Salas Salvadó J., Vizmanos B. (2011). The prevalence of metabolic syndrome in Latin America: A systematic review. Public Health Nutr..

[63] Escobedo J., Schargrodsky H., Champagne B., Silva H., Boissonnet C.P., Vinueza R., Torres M., Hernandez R., Wilson E. (2009). Prevalence of the Metabolic Syndrome in Latin America and its association with sub-clinical carotid atherosclerosis: The CARMELA cross sectional study. Cardiovasc. Diabetol..

[64] Méndez-Hernández P., Flores Y., Siani C., Lamure M., Dosamantes-Carrasco L.D., Halley-Castillo E., Huitrn G., Talavera J.O., Gallegos-Carrillo K., Salmerón J. (2009). Physical activity and risk of Metabolic Syndrome in an urban Mexican cohort. BMC Public Health.

[65] Ervin R.B. (2009). Prevalence of metabolic syndrome among adults 20 years of age and over, by sex, age, race and ethnicity, and body mass index: United States, 2003–2006. Natl. Health Stat. Rep..

[66] Cuevas A., Alvarez V., Carrasco F. (2011). Epidemic of metabolic syndrome in Latin America. Curr. Opin. Endocrinol. Diabetes Obes..

