# Supplementary material for: The Variant rs1784042 of the SIDT2 Gene is Associated with Metabolic Syndrome through Low HDL-c Levels in a Mexican Population
Source: Genes (Basel). 2020 Oct 14;11(10):1192. doi: 10.3390/genes11101192 (PMC7602182; doi:10.3390/genes11101192)
Supplement: Supplementary file 1 [file genes-11-01192-s001.pdf]

Supplementary Table S1. Prevalence of MetS and its components by gender and age categories

Parameters	Age group	Total	Men	Women	*p* Value[1]
Metabolic syndrome-ATP III	18 to 30 years	17.0(11.8-22.2)	20.0(11.0-29.0)	15.1(8.74-21.4)	0.359
	31 to 40 years	34.8(28.8-40.8)	35.8(26.6-44.9)	34.1(26.1-42.1)	0.778
	41 to 50 years	44.7(39.8-49.5)	44.7(36.8-52.5)	44.7(38.4-51.0)	0.997
	51 to 60 years	61.8(57.6-66.0)	61.6(53.0-70.2)	61.9(57.0-66.7)	0.959
	61 to 70 years	68.5(63.8-73.2)	54.5(43.2-65.9)	72.1(67.0-77.2)	0.003
	>70 years	74.6(68.3-80.9)	61.5(45.6-77.5)	78.0(71.3-84.7)	0.035
	Overall	52.6(50.4-54.8)	45.7(41.6-49.7)	55.6(52.9-58.3)	<0.001
Low HDL-c[2]	18 to 30 years	60.7(54.0-67.4)	47.5(36.3-58.7)	69.0(60.9-77.2)	0.002
	31 to 40 years	63.2(57.1-69.2)	58.7(49.3-68.1)	66.7(58.7-74.6)	0.198
	41 to 50 years	63.0(58.3-67.8)	50.9(43.1-58.8)	70.9(65.2-76.6)	<0.001
	51 to 60 years	62.8(58.6-67.0)	55.2(46.4-64.0)	65.2(60.4-70.0)	0.044
	61 to 70 years	57.1(52.1-62.2)	44.2(32.8-55.5)	60.5(54.9-66.0)	0.010
	>70 years	49.2(42.0-56.4)	48.7(32.3-65.1)	49.3(41.2-57.4)	0.945
	Overall	60.2(58.0-62.4)	51.8(47.7-55.8)	63.9(61.4-66.5)	<0.001
High Waist Circumference[3]	18 to 30 years	27.2(21.1-33.3)	12.5(5.09-19.9)	36.5(28.0-45.0)	<0.001
	31 to 40 years	36.8(30.8-42.9)	19.3(11.7-26.8)	50.7(42.3-59.2)	<0.001
	41 to 50 years	47.6(42.7-52.5)	32.1(24.7-39.4)	57.8(51.5-64.0)	<0.001
	51 to 60 years	61.6(57.4-65.8)	31.2(23.0-39.4)	71.4(66.9-75.9)	<0.001
	61 to 70 years	69.8(65.2-74.5)	33.8(23.0-44.6)	79.1(74.4-83.7)	<0.001
	>70 years	68.3(61.6-75.0)	28.2(13.4-43.0)	78.7(72.0-85.3)	<0.001
	Overall	54.1(51.9-56.4)	26.8(23.2-30.4)	66.1(63.5-68.6)	<0.001
High Triglycerides[4]	18 to 30 years	34.5(27.9-41.0)	47.5(36.3-58.7)	26.2(18.4-34.0)	0.002
	31 to 40 years	48.2(41.9-54.5)	52.3(42.8-61.8)	44.9(36.5-53.3)	0.250
	41 to 50 years	55.1(50.2-60.0)	63.5(56.0-71.1)	49.6(43.3-55.9)	0.006
	51 to 60 years	57.7(53.4-62.0)	68.8(60.6-77.0)	54.1(49.1-59.1)	0.004
	61 to 70 years	59.0(54.0-64.0)	55.8(44.5-67.2)	59.8(54.2-65.4)	0.529
	>70 years	51.3(44.1-58.5)	46.2(29.8-62.5)	52.7(44.6-60.7)	0.469
	Overall	53.1(50.9-55.3)	58.2(54.2-62.2)	50.9(48.2-53.5)	0.003
High Blood Pressure[5]	18 to 30 years	13.6(8.87-18.3)	30.0(19.7-40.3)	3.17(0.07-6.28)	<0.001
	31 to 40 years	15.4(10.9-19.9)	22.9(14.9-31.0)	9.42(4.49-14.4)	0.004
	41 to 50 years	29.3(24.8-33.7)	39.0(31.3-46.7)	23.0(17.6-28.3)	<0.001
	51 to 60 years	40.2(35.9-44.4)	49.6(40.7-58.5)	37.1(32.3-41.9)	0.013
	61 to 70 years	58.2(53.2-63.2)	66.2(55.4-77.0)	56.1(50.5-61.8)	0.109
	>70 years	76.2(70.1-82.3)	79.5(66.2-92.7)	75.3(68.4-82.3)	0.587
	Overall	38.9(36.8-41.1)	43.3(39.3-47.3)	37.0(34.5-39.6)	0.010
High Fasting Glucose[6]	18 to 30 years	10.7(6.43-14.9)	18.8(10.0-27.5)	5.56(1.5-9.61)	0.003
	31 to 40 years	28.3(22.7-34.0)	39.4(30.1-48.8)	19.6(12.9-26.3)	<0.001
	41 to 50 years	40.0(35.1-44.8)	50.3(42.5-58.2)	33.2(27.2-39.1)	<0.001
	51 to 60 years	49.7(45.4-54.0)	60.8(52.1-69.5)	46.1(41.2-51.1)	0.004
	61 to 70 years	56.1(51.1-61.1)	59.7(48.5-70.9)	55.1(49.5-60.8)	0.469
	>70 years	51.3(44.1-58.5)	64.1(48.3-79.9)	48.0(39.9-56.1)	0.073
	Overall	42.2(40-44.4.0)	48.4(44.3-52.4)	39.5(36.9-42.1)	<0.001

[1] *p* value was obtained using tests on the equality of proportions. [2] Low HDL-cholesterol: <40 in men and <50 in women; [3] High waist circumference: ≥102 cm in men and ≥88 cm in women, [4] High triglycerides: ≥150 mg/dL, [5] High blood pressure: ≥130/≥85 mmHg, [6] High fasting glucose ≥100 mg/dL or previous T2D diagnosis.

Supplementary Table S2. Top SNPs associated with total cholesterol, HDLc, LDL-c and triglycerides in European and discovery population (pilot HWCS).

Parameters	SNP ID	Chr	Position[1]	Gene	Type of variant	European [3]			HWCS			
						A1[2]	A2	MAF	MAF [4]	β	SE	*p* Value
Cholesterol	rs10501541	11	83189400	*DLG2*	Intronic	C	A	0.222	0.079	-0.053	0.011	2.79E-06
	rs6013184	20	50047098	*NFATC2*	Intronic	A	G	0.100	0.147	-0.040	0.008	4.91E-06
	rs4703842	5	81056637		Intergenic	C	A	0.159	0.293	0.030	0.006	1.24E-05
	rs513783	20	4324820		Intergenic	A	G	0.461	0.419	0.026	0.006	1.65E-05
	rs1029719	17	32550864		Regulatory region	A	G	0.376	0.212	-0.032	0.007	4.14E-05
	rs6787835	3	166538324		Intergenic	G	A	0.464	0.262	0.031	0.007	4.36E-05
	rs13002629	2	124049457		Intergenic	A	G	0.354	0.161	-0.034	0.008	6.06E-05
	rs12597411	16	53060503		Intergenic	A	G	0.219	0.225	0.029	0.007	7.28E-05
	rs2189461	7	8594363	*NXPH1*	Intronic	A	G	0.365	0.347	0.025	0.006	7.56E-05
	rs6460666	7	71006669	*GALNT17*	Intronic	G	A	0.265	0.271	0.028	0.007	7.83E-05
	rs1967398	4	21254536	*KCNIP4*	Intronic	G	A	0.112	0.179	0.032	0.008	7.83E-05
	rs2181165	11	33943644		Regulatory region	A	G	0.435	0.473	-0.025	0.006	8.65E-05
	rs4686969	3	188236356	*LPP*	Intronic	G	A	0.225	0.141	-0.035	0.008	8.95E-05
HDL-c	rs985864	2	104145165		Noncoding transcript	A	C	0.051	0.079	0.059	0.013	1.82E-05
	rs926774	6	47121275		Regulatory region	G	A	0.155	0.435	0.032	0.007	1.93E-05
	rs11060395	12	130008376	*TMEM132D*	Intronic	A	C	0.298	0.156	0.043	0.010	2.45E-05
	rs7594007	2	113351218	*None*	Intronic	G	A	0.454	0.394	0.032	0.007	2.57E-05
	rs6969314	7	38176373		Intergenic	A	G	0.408	0.288	-0.036	0.008	2.77E-05
	rs11694460	2	113355181	*None*	Intronic	A	G	0.453	0.383	0.032	0.007	2.96E-05
	rs2647969	16	51666146	*LOC107984892*	Intronic	C	A	0.364	0.417	-0.032	0.007	3.53E-05
	rs6742861	2	66750152	*MEIS1*	Intronic	G	A	0.322	0.183	-0.040	0.009	4.81E-05

	rs1358018	3	109799304		Intergenic	G	A	0.043	0.128	-0.046	0.011	4.94E-05
	rs10914817	1	34394259	*CSMD2*	Intronic	A	G	0.155	0.115	-0.049	0.012	5.12E-05
	rs2672826	5	177954516	*COL23A1*	Intronic	A	G	0.114	0.063	-0.064	0.015	5.46E-05
	rs777594	8	116361267		Intergenic	A	G	0.384	0.271	-0.033	0.008	6.00E-05
	rs406098	13	48963784		Noncoding transcript	A	G	0.036	0.095	-0.050	0.012	6.11E-05
	rs398158	13	49006265	*RB1*	Intronic	C	A	0.036	0.095	-0.050	0.012	6.11E-05
	rs11889877	2	66080617	*None*	Intronic	A	G	0.167	0.112	0.047	0.011	7.08E-05
	rs2109517	7	116217657	*COMETT*	Intronic	G	A	0.325	0.432	0.030	0.007	7.73E-05
	rs2834383	21	35500111	*MRPS6*	Intronic	G	A	0.092	0.058	-0.062	0.015	9.30E-05
	rs2672818	5	177932674	*COL23A1*	Intronic	A	G	0.168	0.090	-0.051	0.012	9.48E-05
LDL-c	rs3012771	9	134631095		TF binding site	A	G	0.281	0.302	0.042	0.009	7.98E-06
	rs6983236	8	58390733		Intergenic	G	A	0.142	0.106	0.060	0.014	2.26E-05
	rs7186021	16	85486059	*GSE1*	Intronic	A	C	0.466	0.376	-0.035	0.008	3.93E-05
	rs16822576	1	184180060		Regulatory region	C	A	< 0.01	0.425	-0.035	0.008	5.60E-05
	rs4859879	4	78722315	*CNOT6L*	Intronic	A	G	0.294	0.311	0.038	0.009	7.18E-05
	rs1967398	4	21254536	*KCNIP4*	Intronic	G	A	0.112	0.179	0.045	0.011	7.54E-05
	rs355870	2	165631433	*COBLL1*	Intronic	A	G	0.227	0.106	0.059	0.014	7.76E-05
	rs10501541	11	83189400	*DLG2*	Intronic	C	A	0.222	0.079	-0.063	0.015	7.82E-05
	rs6458011	6	37660867	*MDGA1*	Intronic	G	A	0.319	0.201	-0.042	0.010	8.34E-05
	rs13127180	4	55844584		Intergenic	A	G	0.320	0.180	0.045	0.011	8.92E-05
	rs6821744	4	55850984		Intergenic	C	A	0.320	0.180	0.045	0.011	8.92E-05
	rs6823712	4	55855658		Intergenic	A	G	0.321	0.180	0.045	0.011	8.92E-05
	rs9588267	13	111474921		Intergenic	A	G	0.278	0.228	0.041	0.010	9.81E-05
Triglycerides	rs227134	20	8427390	*PLCB1*	Intronic	G	A	0.361	0.122	-0.100	0.022	8.32E-06
	rs2965705	12	11640689	*LOC440084*	Intronic	A	C	0.155	0.290	-0.069	0.015	8.70E-06
	rs1909433	10	1747923	*ADARB2*	Intronic	A	G	0.102	0.217	0.076	0.017	1.08E-05
	rs2427273	20	60881330	*ADRM1*	Synonymous	G	A	0.095	0.110	-0.097	0.022	2.69E-05

rs11250752	10	1750471	*ADARB2*	Intronic	A	G	0.313	0.330	0.063	0.014	2.74E-05
rs7113765	11	97609886		Intergenic	G	A	0.216	0.259	0.068	0.0163	3.42E-05
rs2292741	8	38695996	*TACC1*	Synonymous	G	A	0.152	0.128	0.088	0.0212	3.72E-05
rs929090	22	44313849		Regulatory region	A	G	0.457	0.473	0.058	0.0142	5.00E-05
rs2951754	12	11614777	*LOC440084*	Intronic	C	A	0.071	0.237	-0.068	0.0167	5.20E-05
rs16823373	2	144718006	*GTDC1*	Intronic	A	G	0.040	0.092	-0.096	0.0237	5.28E-05
rs9390235	6	145168455	*UTRN*	Intronic	C	A	0.110	0.082	-0.103	0.0254	5.65E-05
rs11058197	12	125968344	*TMEM132B*	Intronic	A	G	0.285	0.384	0.058	0.0145	6.06E-05
rs3821466	3	125829505	*ALDH1L*	Intronic	A	G	0.298	0.185	-0.075	0.0186	6.34E-05
rs2300932	9	123770436	*C5*	Intronic	A	C	0.392	0.361	0.060	0.0149	6.39E-05
rs6702754	1	154303976	*ATP8B2*	Synonymous	G	A	0.318	0.211	-0.070	0.0174	6.67E-05
rs2248500	8	14612100	*SGCZ*	Intronic	G	A	0.360	0.341	0.057	0.0144	7.74E-05
rs3762182	16	87326113	*LOC101928682*	Intronic	A	G	0.163	0.256	-0.065	0.0165	7.97E-05
rs2147639	13	55581949		Intergenic	G	A	0.301	0.338	-0.059	0.0149	8.74E-05
rs1014415	1	92502060	*EPHX4*	Intronic	G	A	0.236	0.230	-0.065	0.0165	8.96E-05

[1] Chromosomal position (Mb) based on human genome dbSNP build 37; [2] Minor allele; [3] Minor allele frequency (MAF) were identified from GWAS of European populations; [4] MAF estimated from the discovery sample (HWCS population). Only the strongest signals within or near a gene are shown.

Supplementary Table S3. Earlier known gene regions for MetS and related metabolic traits.

SNP ID	Chr	Position[1]	Gene	Type of variant	A1[2]	A2	MAF European[3]	Association	Reference
rs3093059	1	157951759	*CRP*	Intergenic	C	T	0.072	CRP levels	Zhang *et al.*, 2020.
rs17367504	1	11862778	*MTHFR*	Intron	G	A	0.139	Hypertension	Ji *et al.*, 2017.
rs3093077	1	157946259	*CRP*	Intergenic	G	T	0.072	CRP levels	Huang *et al.*, 2019.
rs7561317	2	644953	*TMEM18*	Intergenic	A	G	0.174	Obesity	Jiménez-Osorio *et al.*, 2019.
rs780094	2	27741237	*GCKR*	Intron	T	C	0.411	Lipid metabolism	Wang *et al.*, 2018.
rs146816516	3	152262078	*MBNL1*	Intergenic	G	A	0.000	Adipogenesis	Hung *et al.*, 2020.
rs7638110	3	138903985	*MRPS22*	Intron	A	C	0.075	Obesity	Melka *et al.*, 2012.
rs11242417	5	137599334	*GFRA3*	Intron	C	A	0.162	Cytokine levels	Souza *et al.*, 2010.
rs12678919	8	19844222	*LPL*	Intergenic	G	A	0.119	Lipid metabolism	Xu *et al.*, 2019.
rs10096633	8	19830921	*LPL*	Regulatory	A	G	0.169	Lipid metabolism	Ahmad *et al.*, 2011.
rs2954029	8	126490972	*TRIB1*	Intron	T	A	0.448	Lipid metabolism	Zhang *et al.*, 2019.
rs9644568	8	19928582	*LPL*	Intergenic	A	G	0.146	Lipid metabolism	Quin *et al.*, 2018.
rs328	8	19819724	*LPL*	Stop gained	G	C	0.130	Lipid metabolism	Zhang *et al.*, 2015.
rs10503669	8	19847690	*LPL*	Intergenic	A	C	0.129	Lipid metabolism	Baik *et al.*, 2013.
rs10808546	8	126495818	*TRIB1*	Intron	A	G	0.431	Lipid metabolism	Zhang *et al.*, 2019.
rs76822696	8	84016901	*RALYL*	Intron	A	C	0.289	MetS	Tekola *et al.*, 2015.
rs295	8	19816238	*LPL*	Intron	C	A	0.262	Lipid metabolism	Pirim *et al.*, 2014.
rs17482310	8	19746876	*LPL*	Regulatory	T	G	0.171	Hypertriglyceridemia	Qin *et al.*, 2018.
rs77244975	10	69207975	*CTNNA3*	Intron	C	T	0.000	MetS	Tekola *et al.*, 2015.
rs4590817	10	63467553	*CABCOCO1*	Intron	C	G	0.153	Hypertension	Kawashima *et al.*, 2016.
rs1530440	10	63524591	*CABCOCO1*	Intron	A	G	0.190	Hypertension	Hotta *et al.*, 2012.
rs1784042	11	117065476	*SIDT2*	Intron	A	G	0.424	Total cholesterol/ MetS	Kulminsky *et al.*, 2019 Moon, *et al.*, 2018
rs662799	11	116663707	*APOA5*	Intergenic	G	A	0.083	Dyslipidemia	Wang *et al.*, 2016.
rs651821	11	116662579	*APOA5*	5 prime UTR	T	C	0.083	Hypertension	Wang *et al.*, 2020.
rs964184	11	116648917	*APOA1/ZPR1*	3 prime UTR	G	C	0.162	Lipid metabolism	Qiu *et al.*, 2018.
rs964184	11	116648917	*ZNF259*	3 prime UTR	G	C	0.162	MetS, Obesity, Lipid metabolism,	Qiu *et al.*, 2018.

								Hypertension, Glucose levels	
rs6589567	11	116670676	*APOA5*	Intergenic	A	C	0.133	Lipid metabolism	Au *et al*., 2017.
rs7350481	11	116586283	*BUD13*	Regulatory	A	G	0.098	Lipid metabolism	Yamada *et al*., 2017.
rs11216126	11	116617240	*NR*	Intergenic	C	A	0.238	Triglycerides	Gombojav *et al*., 2016.
rs180349	11	116611827	*NR*	Intergenic	A	C	0.133	MetS	Kim *et al*., 2012.
rs10790162	11	116639104	*BUD13*	Intron	A	G	0.210	Dyslipidemia	Yamada *et al*., 2017.
rs2075290	11	116653296	*ZNF259/ZPR1*	Intron	C	T	0.092	MetS	Jasim *et al*., 2018.
rs2266788	11	116660686	*APOA5/ZPR1*	3 prime UTR	G	A	0.091	TG, VLDL	Kraja *et.al*., 2011.
rs10849915	12	111333622	*CCDC63*	Intron	C	T	0.348	MetS	Lee *et al*., 2018.
rs11065756	12	111338794	*CCDC63*	Intron	T	C	0.076	MetS	Lee *et al*., 2018.
rs7964157	12	117982220	*KSR2*	Intron	T	C	0.404	MetS	Tekola *et al*., 2015.
rs671	12	112241766	*ALDH2*	Missense	A	G	0.000	Hypertension	Chen *et al*., 2018.
rs16940170	15	58684282	*LOC101928635*	Intron	A	G	0.153	Cholesterol	Lee *et al*., 2018.
rs16940212	15	58694020	*LOC101928635*	Intron	T	G	0.200	Cholesterol	Lee *et al*., 2018.
rs495348	15	58687790	*LOC101928635*	Intron	C	G	0.156	Cholesterol	Lee *et al*., 2018.
rs6511720	19	11202306	*LDLR*	Intron	A	C	0.110	Lipid metabolism	Fairoozy *et al*., 2016.
rs12594515	15	45985071	*SQOR*	Intergenic	C	G	0.472	Obesity	Cai *et al*., 2018.
rs8050136	16	53816275	*FTO*	Intron	A	C	0.414	Obesity and Type 2 diabetes	Bego *et al*., 2019.
rs173539	16	56988044	*CETP*	Intergenic	T	C	0.298	MetS	Oh *et al*., 2020.
rs16944558	18	442441	*COLEC12*	Intron	T	C	0.092	MetS	Lin *et al*., 2017.

[1] Chromosomal position (Mb) based on human genome dbSNP build 37; [2] Minor allele; [3] Minor allele frequency (MAF) estimated from the European population. The genes were identified using NHGRI-EBI GWAS catalog (https://www.ebi.ac.uk/gwas) and PubMed search. In bold, the genetic variant located in *SIDT2* gene is shown, which we identified and selected in the pilot GWAS for replication in the total sample.

Supplementary Figure S4. Genetic region on chromosome 11 associated with HDL-c in pilot GWAS of BMD and related metabolic traits.

SNP ID	Chr	Position[1]	Gene	Type of variant	A1[2]	A2	MAF	β	SE β	*p* Value
rs10400366	11	117000955		Intergenic	G	A	0.371	1.268	0.399	0.002
rs2735181	11	117009789		Intergenic	C	T	0.370	1.273	0.401	0.002
rs2622934	11	117014862		Intergenic	G	T	0.370	1.273	0.401	0.002
rs2735183	11	117015195	*PAFAH1B2*	Intronic	C	T	0.370	1.273	0.401	0.002
rs4938351	11	117022352	*PAFAH1B2*	Intronic	A	C	0.370	1.273	0.401	0.002
rs10892080	11	117035502	*PAFAH1B2*	Intronic	A	G	0.367	1.275	0.399	0.002
rs6589602	11	117037065	*PAFAH1B2*	Intronic	T	C	0.367	1.275	0.399	0.002
rs10750103	11	117042238	*PAFAH1B2*	Intronic	C	T	0.366	1.254	0.399	0.002
rs10892083	11	117042580	*PAFAH1B2*	Intronic	G	C	0.309	1.075	0.414	0.010
rs7120565	11	117042949	*PAFAH1B2*	Intronic	T	C	0.366	1.254	0.399	0.002
rs7925256	11	117045335	*PAFAH1B2*	Intronic	T	C	0.162	1.627	0.538	0.003
rs1784042	*11*	*117065476*	*SIDT2*	*Intronic*	G	*A*	*0.308*	*1.134*	*0.414*	*0.006*
rs652455	11	117071449	*TAGLN*	Intronic	C	T	0.216	1.362	0.478	0.005
rs2269396	11	117078378	*PCSK7*	UTR3	C	T	0.295	1.387	0.430	0.001
rs236915	11	117088450	*PCSK7*	Intronic	A	G	0.362	-1.266	0.402	0.002
rs74830	11	117090558	*PCSK7*	Intronic	C	T	0.362	-1.274	0.402	0.002
rs236919	11	117095361	*PCSK7*	Intronic	C	T	0.354	-1.170	0.402	0.004
rs500389	11	117100594	*PCSK7*	Intronic	A	G	0.335	-1.117	0.403	0.006
rs117524108	11	117115763	*RNF214*	Intronic	G	A	0.076	-2.121	0.743	0.005

[1] Chromosomal position (Mb) based on human genome dbSNP build 37; [2] Minor allele. Only the strongest signals within or near a gene are shown. The variant rs1784042, which was located within the *SIDT2* gene on chromosome 11, was the strongest signal (pGWAS = 0.006) for HDL-c.

Supplementary Table S5. Allele frequencies of *SIDT2* gene polymorphisms

	Minor allele frequency	
**Population**	**rs17120425 (%)**	**rs1784042 (%)**
HWCS[1]	10.0	28.8
MXL[2,3]	6.0	28.0
YRIb[4]	0.5	1.9
CHBb[5]	1.0	19.0
CEUb[6]	0.0	46.0

[1]HWCS, Health workers cohort study; [2] Data from 1000 Genomes; [3]MXL, Mexicans from Los Angeles; [4]YRI, Yoruba in Ibadan, Nigeria; [5]CHB, Chinese Han from Beijing and [6]CEU, Northern and Western Europeans from Utah (Caucasians). p value for EHW was < 0.05.

Supplementary Table S6. Association of rs17120425 polymorphism and MetS by postmenopausal status

		Premenopausal				Postmenopausal			
**Outcome**	**Genotype**	**Control, n(%)**	**Case, n(%)**	**OR[1,6] (95% CI)**	***p* Value**	**Control, n(%)**	**Case, n(%)**	**OR[1] (95% CI)**	***p* Value**
MetS [2]	GG	305(79.6)	197(79.8)	Ref.		181(81.2)	429(83.6)	Ref.	
	GA	76(19.9)	45(18.2)	0.82(0.53-1.25)	0.356	39(17.5)	79(15.4)	0.88(0.57-1.35)	0.555
	AA	2(0.5)	5(1.9)	2.57(0.46-14.4)	0.282	3(1.4)	5(1.0)	1.03(0.20-5.17)	0.974
	Additive model			0.94(0.64-1.38)	0.748			0.91(0.62-1.33)	0.616
Low HDL-c [3]	GG	142(73.2)	360(82.6)	Ref.		169(75.1)	441(86.3)	Ref.	
	GA	50(25.8)	71(16.3)	0.54(0.36-0.82)	0.004	53(23.6)	65(12.7)	0.45(0.30-0.68)	$1x10^{-4}$
	AA	2(1.0)	5(1.2)	1.00(0.19-5.28)	0.996	3(1.3)	5(1.0)	0.65(0.15-2.78)	0.564
	Additive model			0.62(0.42-0.90)	0.012			0.52(0.36-0.75)	$4x10^{-4}$
Impaired glucose tolerance [4]	GG	376(81.7)	91(75.8)	Ref.		307(84.1)	190(84.8)	Ref.	
	GA	81(17.6)	26(21.7)	1.24(0.74-2.09)	0.407	53(14.5)	33(14.7)	1.01(0.63-1.61)	0.976
	AA	3(0.7)	3(2.5)	3.00(0.55-16.20)	0.202	5(1.4)	1(0.5)	0.32(0.04-2.75)	0.299
	Additive model			1.36(0.86-2.14)	0.184			1.46(0.95-2.24)	0.083
Type 2 Diabetes [5]	GG	376(81.7)	35(70.0)	Ref.		307(84.1)	113(76.9)	Ref.	
	GA	81(17.6)	14(28.0)	1.69(0.83-3.42)	0.145	53(14.5)	32(21.8)	1.70(1.04-2.78)	0.034
	AA	3(0.7)	1(2.0)	2.05(0.18-22.99)	0.560	5(1.4)	2(1.4)	1.01(0.19-5.28)	0.995
	Additive model			1.58(0.86-2.93)	0.142			1.48(0.96-2.28)	0.072

[1] Model adjusted for age and family group. [2] MetS: Metabolic Syndrome (ATP-III definition). [3] Low HDL-cholesterol: <40 in men and <50 in women; [4] Impaired glucose tolerance: >100 to <126 glucose levels; [5] Type 2 diabetes: ≥126 glucose levels or self-report of physician diagnosis. [6] OR: Odds ratio

Supplementary Table S7. Association between rs17120425 and HDL-c levels

			Total		Men		Women		Premenopausal		Postmenopausal	
**Outcome**	**Genotype**	**n(%)**	**β (95% CI) [1]**	***p* Value**	**β (95% CI) [2]**	***p* Value**	**β (95% CI) [2]**	***p* Value**	**β (95% CI) [2]**	***p* Value**	**β (95% CI) [2]**	***p* Value**
HDL-c (mg/dL)	GG	1600 (81.9)	Ref.		Ref.		Ref.		Ref.		Ref.	
	GA	333 (17.0)	3.76(2.49,5.02)	$7.0x10^{-9}$	2.77(0.71,4.84)	0.009	4.17(2.59,5.74)	$2.4x10^{-7}$	3.19(1.10,5.29)	0.003	5.09(2.77,7.41)	$2x10^{-5}$
	AA	21(1.1)	4.26(-0.32,8.84)	0.068	2.35(-5.13,9.83)	0.538	5.08(-0.62,10.77)	0.08	5.06(2.80,12.92)	0.206	4.74(-3.47,12.94)	0.257
	Additive model		3.39(2.26,4.52)	$4.5x10^{-9}$	2.42(0.58,4.26)	0.01	3.81(2.40,5.21)	$1.3x10^{-7}$	3.06(1.17,4.94)	0.002	4.46(2.40,6.51)	$2x10^{-5}$

[1] Model adjusted for age, sex and family group. [2] Model adjusted for age and family group.

Supplementary Table S8. Association of rs1784042 polymorphism and MetS by postmenopausal status

		Premenopausal				Postmenopausal			
**Outcome**	**Genotype**	**Control, n(%)**	**Case, n(%)**	**OR[1,6] (95% CI)**	***p* Value**	**Control, n(%)**	**Case, n(%)**	**OR[1] (95% CI)**	***p* Value**
MetS [2]	GG	192(50.3)	134(54.7)	Ref.		102(45.3)	262(51.0)	Ref.	
	GA	157(41.1)	96(39.2)	0.81(0.57-1.15)	0.247	99(44.0)	204(39.7)	0.77(0.55-1.08)	0.135
	AA	33(8.6)	15(6.1)	0.69(0.35-1.34)	0.271	24(10.7)	48(9.3)	0.66(0.38-1.15)	0.140
	Additive model			0.82(0.63-1.07)	0.151			0.80(0.63-1.02)	0.068
Low HDL-c [3]	GG	86(44.6)	240(55.3)	Ref.		98(43.0)	266(52.1)	Ref.	
	GA	86(44.6)	167(38.5)	0.68(0.47-0.97)	0.034	106(46.5)	197(38.6)	0.67(0.48-0.94)	0.02
	AA	21(10.9)	27(6.2)	0.46(0.24-0.85)	0.014	24(10.5)	48(9.4)	0.74(0.43-1.27)	0.273
	Additive model			0.68(0.52-0.88)	0.004			0.79(0.62-1.00)	0.048
Impaired glucose tolerance [4]	GG	238(52.0)	61(51.3)	Ref.		171(46.7)	117(52.0)	Ref.	
	GA	184(40.2)	47(39.5)	0.96(0.63-1.52)	0.912	158(43.2)	88(39.1)	0.82(0.58-1.16)	0.264
	AA	36(7.9)	11(9.2)	1.27(0.58-2.71)	0.537	37(10.1)	20(8.9)	0.79(0.44-1.43)	0.437
	Additive model			0.81(0.50-1.32)	0.401			0.86(0.67-1.11)	0.253
Type 2 Diabetes [5]	GG	238(52.0)	27(54.0)	Ref.		171(46.7)	76(51.4)	Ref.	
	GA	184(40.2)	22(44.0)	1.03(0.55-1.93)	0.927	158(43.2)	57(38.5)	0.81(0.54-1.22)	0.309
	AA	36(7.9)	1(2.0)	0.27(0.03-2.11)	0.212	37(10.1)	15(10.1)	0.89(0.46-1.72)	0.72
	Additive model			0.82(0.49-1.37)	0.448			0.89(0.67-1.19)	0.436

[1] Model adjusted for age and family group. [2] MetS: Metabolic Syndrome (ATP-III definition). [3] Low HDL-cholesterol: <40 in men and <50 in women; [4] Impaired glucose tolerance: >100 to <126 glucose levels; [5] Type 2 diabetes: ≥126 glucose levels or self-report of physician diagnosis. [6] OR: Odds ratio

Supplementary Table S9. Association between rs1784042 and components of the MetS

		**Total**		**Men**		**Women**		**Premenopausal**		**Postmenopausal**	
**Outcome**	**Genotype**	**β (95% CI) [1]**	***p* Value**	**β (95% CI) [2]**	***p* Value**	**β (95% CI) [2]**	***p* value**	**β (95% CI) [2]**	***p* Value**	**β (95% CI) [2]**	***p* Value**
Triglycerides levels (mg/dL)	GG	Ref.		Ref.		Ref.		Ref.		Ref.	
	GA	-15.54(-25.95-5.12)	0.003	-17.50(-41.77-6.77)	0.157	-14.76(-25.33-4.18)	0.006	-9.66(-23.14,3.83)	0.160	-19.83(-35.35-4.32)	0.012
	AA	-23.39(-42.17-4.60)	0.015	-55.44(-103.43-7.45)	0.024	-12.26(-30.67,6.14)	0.191	1.91(-22.95,26.77)	0.880	-18.61(-44.34-7.12)	0.156
	Additive model	-13.35(-21.14-5.56)	0.001	-22.69(-41.62-3.75)	0.019	-9.65(-17.42-1.87)	0.015	-3.79(-14.00,6.38)	0.465	-13.24(-24.38-2.09)	0.020
HDL-c levels (mg/dL)	GG	Ref.		Ref.		Ref.		Ref.		Ref.	
	GA	1.16(0.16,2.17)	0.023	0.68(-0.89,2.24)	0.397	1.37(0.10,2.64)	0.035	1.50(-0.23,3.22)	0.089	1.61(-0.21,3.43)	0.083
	AA	3.31(1.50,5.12)	3x10^{-4}	3.26(0.17,6.36)	0.039	3.36(1.14,5.58)	0.003	6.02(2.83,9.20)	0.0003	1.59(-1.43,4.60)	0.302
	Additive model	1.44(0.69,2.19)	2x10^{-4}	1.16(-0.06,2.38)	0.063	1.55(0.62,2.48)	0.001	2.33(1.03,3.63)	0.0005	1.10(-0.21,2.40)	0.100
LDL-c levels (mg/dL)	GG	Ref.		Ref.		Ref.		Ref.		Ref.	
	GA	3.79(0.53,7.05)	0.023	3.28(-2.35,8.92)	0.253	3.96(-0.007,7.93)	0.050	2.77(-2.41,7.96)	0.294	4.61(-1.10,10.32)	0.113
	AA	3.10(-2.74,8.94)	0.298	9.56(-1.45,20.56)	0.089	1.05(-5.83,7.92)	0.765	1.09(-8.42,10.59)	0.823	2.90(-6.53,12.32)	0.547
	Additive model	2.51(0.08,4.94)	0.043	4.05(-0.32,8.43)	0.070	1.91(-1.00,4.82)	0.198	1.53(-2.36,5.42)	0.440	2.62(-1.47,6.71)	0.209
Waist circumference (cm)	GG	Ref.		Ref.		Ref.		Ref.		Ref.	
	GA	-0.73(-1.78,0.32)	0.172	-0.35(-2.10,1.39)	0.690	-0.89(-2.19,0.41)	0.178	-0.97(-2.90-0.96)	0.324	-0.87(-2.58,0.85)	0.322
	AA	-2.45(-4.34,-0.57)	0.011	-0.52(-3.96,2.92)	0.767	-3.09(-5.33-0.84)	0.007	-5.41(-9.0-1.87)	0.003	-0.99(-3.84,1.85)	0.492
	Additive model	-1.01(-1.80,-0.23)	0.011	-0.31(-1.67,1.05)	0.658	-1.28(-2.23-0.33)	0.008	-1.94(-3.39,0.48)	0.009	-0.63(-1.87,0.60)	0.311

[1] Model adjusted for age, sex and family group. [2] Model adjusted for age and family group.

Supplementary Table S10. Conditional analysis for rs17120425 and MetS and its components in additive models

	Total		Men		Women	
Outcome	OR[1,7] (95% CI)	*p* Value	OR[2] (95% CI)	*p* Value	OR[2] (95% CI)	*p* Value
MetS[3]	0.84 (0.66-1.08)	0.182	0.61 (0.38-0.99)	0.044	0.95 (0.71-1.28)	0.744
Low HDL-c [4]	0.57 (0.45-0.72)	4.0×10^{-6}	0.62 (0.39-0.97)	0.038	0.54 (0.41-0.72)	2.0×10^{-4}
Impaired glucose tolerance [5]	0.99 (0.75-1.32)	0.965	0.72 (0.43-1.23)	0.231	1.11 (0.79-1.56)	0.552
Type 2 Diabetes [6]	1.42 (1.01-1.99)	0.044	0.68 (0.34-1.34)	0.263	1.80 (1.21-2.67)	0.004

[1] Model adjusted for age, sex, family group and rs1784042. [2] Model adjusted for age, family group and rs1784042. [3] MetS: Metabolic Syndrome (ATP-III definition). [4] Low HDL-cholesterol: <40 in men and <50 in women; [5] Impaired glucose tolerance: >100 to <126 glucose levels; [6] Type 2 diabetes: ≥126 glucose levels or self-report of physician diagnosis. [7] OR: Odds ratio

Supplementary Table S11. Conditional analysis for rs1784042 and MetS and its components in additive models

	Total		Men		Women	
Outcome	OR[1,7] (95% CI)	*p* Value	OR[2] (95% CI)	*p* Value	OR[2] (95% CI)	*p* Value
MetS[3]	0.89 (0.94-1.44)	0.152	1.00 (0.73-1.36)	0.996	0.86 (0.71-1.05)	0.141
Low HDL-c [4]	0.90 (0.77-1.06)	0.214	0.95 (0.70-1.28)	0.735	0.88 (0.73-1.07)	0.197
Impaired glucose tolerance [5]	0.94 (0.78-1.13)	0.498	1.08 (0.77-1.52)	0.663	0.91 (0.73-1.13)	0.386
Type 2 Diabetes [6]	0.88 (0.70-1.12)	0.304	1.41 (0.91-2.20)	0.129	0.74 (0.55-0.99)	0.042

[1]Model adjusted for age, sex, family group and rs1784042. [2]Model adjusted for age, family group and rs17120425. [3]MetS: Metabolic Syndrome (ATP-III definition). [4] Low HDL-cholesterol: <40 in men and <50 in women; [5] Impaired glucose tolerance: >100 to <126 glucose levels; [6] Type 2 diabetes: ≥126 glucose levels or self-report of physician diagnosis. [7] OR: Odds ratio

Supplementary Table S12. Haplotype Association Analysis of *SIDT2* gene SNPs and MetS and its components

		Total		Men		Women	
Outcome	Haplotype	OR[1,7] (95% CI)	*p* Value	OR[2] (95% CI)	*p* Value	OR[2] (95% CI)	*p* Value
MetS [3]	GG	Ref.		Ref.		Ref.	
	GA	0.93 (0.79-1.09)	0.347	1.04 (0.77-1.39)	0.814	0.87 (0.72-1.06)	0.163
	AG	0.70 (0.36-1.36)	0.296	0.32 (0.04-2.87)	0.308	0.70 (0.34-1.42)	0.321
	AA	0.84 (0.66-1.06)	0.132	0.66 (0.43-1.02)	0.063	0.92 (0.70-1.22)	0.562
Low HDL-c [4]	GG	Ref.		Ref.		Ref.	
	GA	0.92 (0.79-1.09)	0.347	0.95 (0.71-1.28)	0.748	0.90 (0.73-1.09)	0.280
	AG	0.86 (0.44-1.67)	0.651	0.62 (0.10-3.73)	0.600	0.80 (0.38-1.66)	0.541
	AA	0.52 (0.41-0.65)	2.9×10^{-8}	0.60 (0.39-0.92)	0.019	0.48 (0.36-0.63)	3×10^{-6}
Impaired glucose tolerance [5]	GG	Ref.		Ref.		Ref.	
	GA	0.96 (0.80-1.16)	0.699	1.09 (0.78-1.51)	0.609	0.94 (0.75-1.17)	0.566
	AG	1.25 (0.58-2.66)	0.568	1.62 (0.23-11.60)	0.631	1.29 (0.56-2.94)	0-551
	AA	0.92 (0.70-1.21)	0.551	0.73 (0.45-1.19)	0.206	1.03 (0.74-1.44)	0.863
Type 2 Diabetes [6]	GG	Ref.		Ref.		Ref.	
	GA	0.95 (0.75-1.21)	0.673	1.57 (1.05,2.36)	0.029	0.75 (0.55-1.01)	0.057
	AG	1.58 (0.65-3.82)	0.311	-		1.43 (0.55-3.72)	0.459
	AA	1.30 (0.94-1.78)	0.109	1.04 (0.57-1.90)	0.898	1.44 (0.99-2.08)	0.051

[1] Model adjusted for age, sex and family group. [2] Model adjusted for age and family group. [3] MetS: Metabolic Syndrome (ATP-III definition). [4] Low HDL-cholesterol: <40 in men and <50 in women; [5] Impaired glucose tolerance: >100 to <126 glucose levels; [6] Type 2 diabetes: ≥126 glucose levels or self-report of physician diagnosis. [7] OR: Odds ratio

Supplementary Figure S1. LocusZoom plot showing the HDL-c associated region.

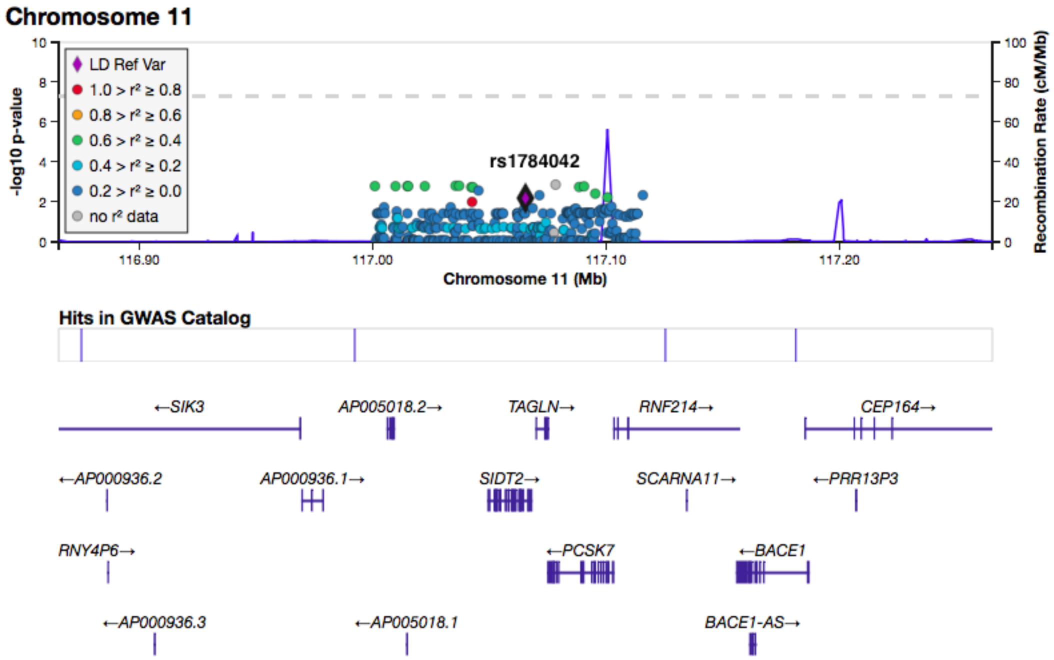

The x-axis is the physical position on the chromosome (Mb), and the y-axis denotes the association test result as the −log (*p* value). SNPs are color-coded according to the linkage disequilibrium (LD) with the lead SNP rs1784042 (purple diamond).

Supplementary Figure S2. HDL-c levels by genotypes of *SIDT2* gene variants.

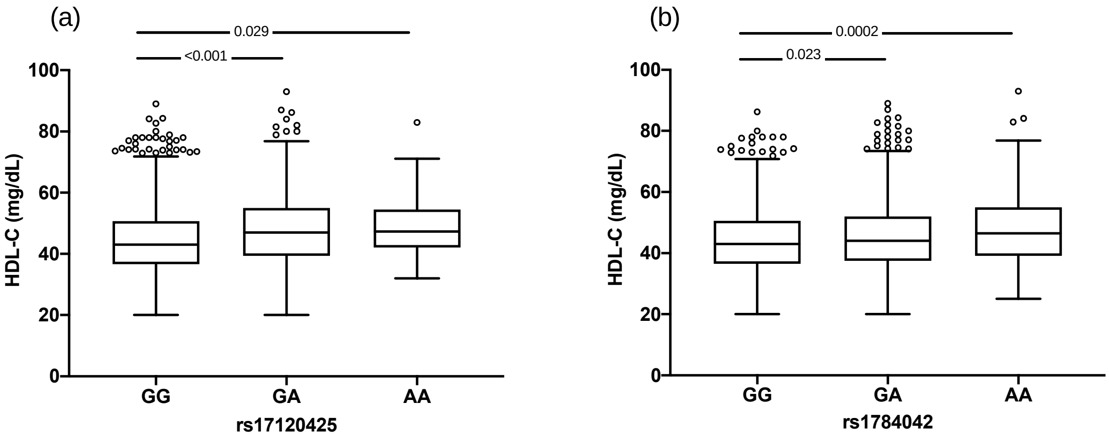

(a) HDL-c levels by genotypes of rs17120425; (b) HDL-c levels by genotypes of rs1784042. p value < 0.05 is considered statistically significant.

Supplementary Figure S3. Prevalence of low HDL-c by genotypes of *SIDT2* gene variants.

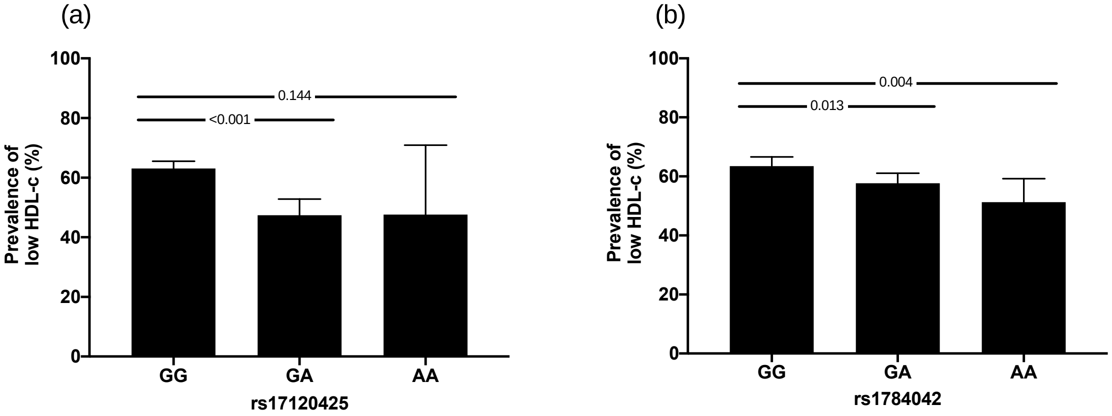

(a) Prevalence of low HDL-c by genotypes of rs17120425; (b) Prevalence of low HDL-c by genotypes of rs1784042. p value < 0.05 is considered statistically significant.

Supplementary Figure S4. Prevalence of high waist circumference by genotypes of *SIDT2* gene variants.

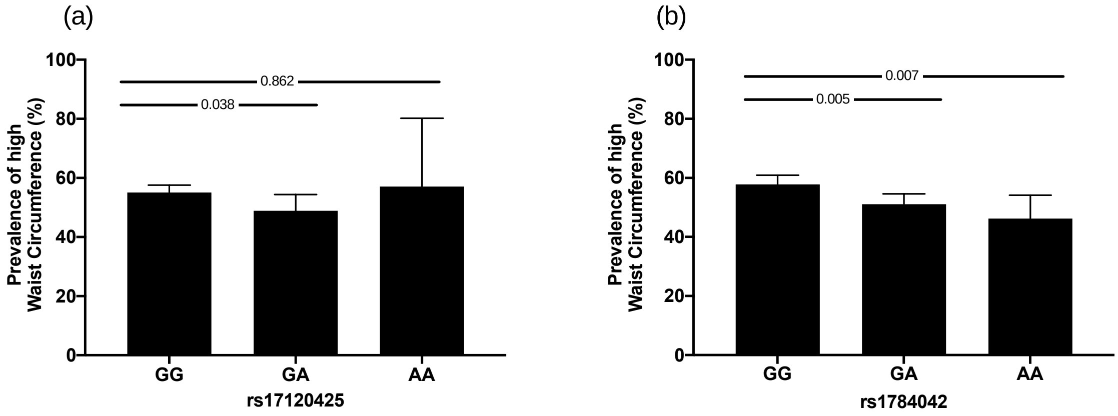

(a) Prevalence of high waist circumference by genotypes of rs17120425; (b) Prevalence of high waist circumference by genotypes of rs1784042. p value < 0.05 is considered statistically significant.

Supplementary Figure S5. Pairwise linkage disequilibrium (LD) between the two *SIDT2* variants associated with MetS and HDL-c, in the present study.

(a) (b)

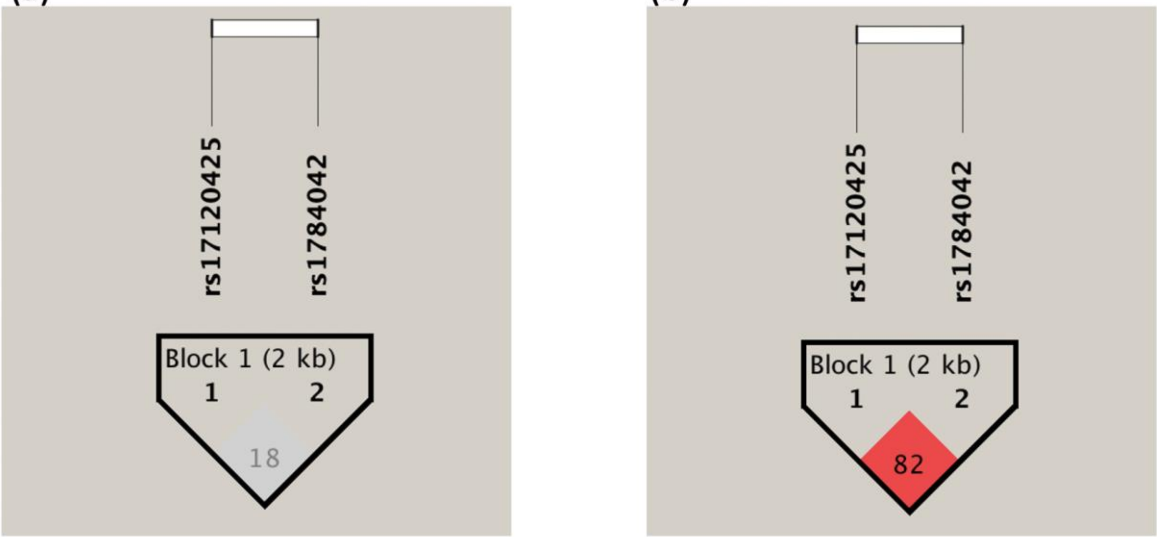

(a) Left panel represents the LD plot with r2 values and (b) right panel represents the LD plot with D′ values.
